# Intestinal epithelial Smad7 drives purine metabolic dysregulation and ileal inflammation

**DOI:** 10.1186/s12929-026-01224-3

**Published:** 2026-02-17

**Authors:** Federica Laudisi, Mattia Alberto Serra, Lorenzo Tomassini, Massimo Claudio Fantini, Gustavo Monasterio, Ning He, Eduardo Maria Sommella, Angela Ortenzi, Giuseppe Sigismondo Sica, Francesca Zorzi, Teresa Pacifico, Ombretta Melaiu, Cristiano De Stefanis, Valentina D’Oria, Carmine Stolfi, Ivan Monteleone, Eduardo Javier Villablanca, Giovanni Monteleone

**Affiliations:** 1 Department of Systems Medicine, University of Rome Tor Vergata, Rome, Italy; 2 Department of Medical Science and Public Health, University of Cagliari, Cagliari, Italy; 3 Division of Immunology and Respiratory Medicine, Department of Medicine Solna (MedS), Karolinska Institute and University Hospital; SE-171 76 Stockholm, Sweden; Center for Molecular Medicine (CMM), Karolinska University Hospital, SE-171 64 Solna, Sweden; 4 Department of Pharmacy, University of Salerno, Fisciano, 84084 Salerno, Italy; 5 Department of Surgery, University of Rome Tor Vergata, Rome, Italy; 6 Gastroenterology Unit, Azienda Ospedaliera Policlinico Tor Vergata, Rome, Italy; 7 Department of Clinical Sciences and Translational Medicine, University of Rome Tor Vergata, Rome, Italy; 8 Bambino Gesù Children’s Hospital, IRCCS, Rome, Italy; 9 Department of Biomedicine and Prevention, University of Rome Tor Vergata, Rome, Italy

**Keywords:** Crohn’s disease, Ulcerative colitis, IBD, TGF-*β*, Mucosal immunity

## Abstract

**Background and aims:**

Defects of the gut epithelial barrier and counter-regulatory mechanisms are a prerequisite for the onset of intestinal inflammation in patients with inflammatory bowel diseases (IBD). Elevated levels of Smad7, an inhibitor of TGF-β1, in both epithelial and immune cells have been associated with the pathological processes observed in IBD. We aim to determine the relevance of Smad7 expression in the epithelial compartment.

**Methods:**

We generated mice overexpressing Smad7 in the gut epithelium (Smad7TgCre^+^) and monitored pathology development. Inflammatory cell infiltration was assessed using multiplex immunofluorescence and flow cytometry. To investigate the molecular mechanisms underlying ileal damage, we performed spatial transcriptomics on frozen ileal samples and metabolomics on intestinal epithelial cells (IECs) from Smad7TgCre^+^ and control mice. The impact of adenosine on Mucin-2 expression was evaluated through both in vitro and in vivo approaches. Finally, we evaluated the protein expression of CD73 and Smad7 in ileal tissue from Crohn’s disease (CD) patients.

**Results:**

Smad7TgCre^+^ mice developed spontaneous terminal ileitis, which was characterized by villus shortening and widening, mucus depletion, increased intestinal permeability, and infiltration of CD8 + T cells. By integrating spatial transcriptomics and metabolomics data, we demonstrated that Smad7TgCre^+^ mice exhibited altered expression of enzymes involved in purine metabolism, particularly CD73, the key enzyme responsible for adenosine production. Consequently, these mice produced lower levels of adenosine, and oral adenosine administration restored mucus production and alleviated the ileal pathology. Lastly, we observed that in the inflamed ileum of CD patients, elevated Smad7 levels were correlated with decreased CD73 expression.

**Conclusion:**

Our data indicate that Smad7 overexpression in IECs is sufficient to promote ileal mucosal damage, which is linked to the impairment of purine metabolism.

**Supplementary Information:**

The online version contains supplementary material available at 10.1186/s12929-026-01224-3.

## Introduction

Ulcerative colitis (UC) and Crohn’s disease (CD), the major inflammatory bowel disease (IBD) subtypes, are chronic inflammatory disorders of the gastrointestinal tract [1]. The etiology of IBD is unknown, but circumstantial evidence indicates that both UC and CD are multifactorial pathologies, with contributions from host genetics, environment, and intestinal microbiota, and characterized by an excessive mucosal immune response that is promoted by defects in the gut epithelial barrier and enhanced translocation of luminal microorganisms to the lamina propria [2–7]. Both genetic and experimental observations also suggest that the amplification and maintenance of such detrimental immune responses are linked to a defective expression and/or function of counter-regulatory mechanisms [8]. One such mechanism involves the production and function of immunosuppressive molecules, such as interleukin (IL)-10. Indeed, IL-10–deficient mice spontaneously develop chronic intestinal inflammation, and loss-of-function variants in the IL-10 receptor contribute to the development of very-early-onset IBD [9–11]. Similarly, mice with defects in the production or function of TGF-β1, another regulatory cytokine, develop gut inflammation, and individuals with Loeys-Dietz syndrome, a pathology caused by heterozygous mutations in the genes encoding the subunits of the TGF-β1 receptor, have an increased risk of IBD as compared with the general population [12–15].

TGF-β1 functions are mediated by a receptor complex, composed of two type I TGF-β receptors and two type II TGF-β receptors, which phosphorylates and activates Smad2/3 molecules after binding the cytokine [16]. Once activated, Smad2/3 interact with Smad4, and the complex moves to the nucleus to control gene expression in several ways (e.g., binding to Smad-responsive regulatory regions, regulation of mRNA splicing, stability, and translation) [17]. The TGF-β1/Smad signaling pathway is negatively controlled by Smad7, an intracellular protein that interacts with the activated TGF-βRI, thus preventing the coupling and phosphorylation of Smad2/3 [18, 19]. Additionally, Smad7 inhibits TGF-β1 function by promoting TGF-β receptor dephosphorylation and degradation, and by preventing the interaction of the Smad2/3/4 complex with DNA [18, 19].

The ability of TGF-β1 to maintain mucosal homeostasis is linked to its regulatory effects on multiple steps of innate and adaptive immunity [20]. In the gut, TGF-β1 targets also epithelial cells, thereby eliciting signals that promote barrier integrity and repair [21].

Studies characterising the expression and signalling of TGF-β1 during gut inflammation have shown that the cytokine is produced in excess in the inflamed gut of IBD patients as well as in the inflamed colons of mice with IBD-like experimental colitis [22, 23]. Nonetheless, in both IBD and mouse experimental colitides, there is a defective activity of TGF-β1, which is due to enhanced expression of Smad7 [24, 25]. Consistently, inhibition of Smad7 restores TGF-β1 activity, with the downstream effect of suppressing many inflammatory pathways and attenuating the ongoing gut inflammation [23, 25]. Analysis of Smad7 cell sources in IBD mucosa revealed that the protein is over-expressed not only in immune cells but also in intestinal epithelial cells (IECs) [26].

To determine the impact of Smad7 overexpression in epithelial cells on gut homeostasis, we generated a mouse that overexpresses Smad7 selectively in the gut epithelium and monitored the development of pathology, both spontaneously and after treatment with compounds that induce epithelial damage and gut inflammation. We also assessed the molecular events driving gut damage in this model.

## Material and methods

### Animals

Smad7TgCre^+^ mice were generated by crossing VillinCre^ERT2^-positive mice (kindly provided by S. Vetrano, Milan, Italy) with Smad7Tg mice (Genoway, Lyon, France) [27]. Conditional overexpression of Smad7 gene in IECs (Villin-positive cells) of Smad7TgCre^+^ mice was obtained by intraperitoneal injections of tamoxifen (TMX; 1 mg/mouse, #T5648 Sigma, St Louis, Missouri, USA) for 5 consecutive days. Smad7Tg mice were used as controls. Mice were then sacrificed 4 weeks after the last TMX injection, and both the ileum and colon samples were harvested. Mice were housed in the University of Rome ‘Tor Vergata’ animal facility (Rome, Italy) and Plaisant animal facility (Castel Romano, Rome). Female mice, aged 7–8 weeks, were used for all experiments.

All in vivo experimental procedures were approved by the relevant animal ethics committee in accordance with Italian legislation on animal experimentation (Authorization Number A69A0.69) and conducted in compliance with European Directive 2010/63/EU.

In some experiments, one week after the final TMX treatment, Smad7TgCre^+^ and control mice were subcutaneously injected with indomethacin (5mg/Kg, #I7378; Sigma) and sacrificed after 24 h. In other experiments, Smad7TgCre^+^ and control mice were treated with Propyzamide (100 mg/kg, #45,645, Sigma) by oral gavage every other day and sacrificed after 2 weeks. Ileal samples were then collected for the histologic analysis.

Additionally, in a separate set of experiments, the day following the last TMX treatment, Smad7TgCre^+^ mice were treated with adenosine (10 mg/kg, #A4036, Sigma) or vehicle (control) by oral gavage every other day until day 28.

CD8⁺ cell depletion was achieved by intraperitoneal injection of Smad7TgCre^+^ mice with an anti-mouse CD8 antibody (5 mg/kg; #100,765, BioLegend, San Diego, California, USA) four times, beginning the day after the final TMX injection and subsequently every three days. Splenocytes were then isolated and analyzed by flow cytometry.

To deplete gut microbiota, Smad7TgCre^+^ and control mice were treated with a cocktail of antibiotics (vancomycin #V2002, ampicillin #A0166, neomycin #N1876, and metronidazole #M3761, 1 g/L each, Sigma) two weeks after the final TMX injection. Mice were sacrificed on day 28, and ileal samples were collected for analysis.

### Histopathology scoring

Ileal and colonic tissues from Smad7TgCre^+^ and control mice were cut longitudinally, and luminal contents were removed. Beginning from the distal end (i.e., rectum) with the luminal side facing upward, the ileum and colon were rolled to form a Swiss roll, positioning the distal portion at the center and the proximal portion on the outer edge. The Swiss roll was placed in a histology cassette and snap-frozen for 1 min in liquid nitrogen–cooled isopentane. The frozen tissue was then embedded in Optimal Cutting Temperature (OCT) compound (#6502, Epredia, Kalamazoo, MI, USA) on dry ice and stored at −80 °C. For histopathological analysis, sections were stained with hematoxylin and eosin and scored in a blinded manner based on epithelial alterations and immune cell infiltration, as previously described [28, 29].

### Patients

Intestinal biopsies were obtained from inflamed and non-inflamed ileal mucosa of five CD patients undergoing surgery for chronic active disease unresponsive to medical treatment (n = 4) or stricturing disease (n = 1). Samples were embedded in OCT on dry ice and stored at −80 °C. The study was approved by the local ethics committee (Protocol Number: R.S. 58.23), and all patients provided written informed consent.

### Confocal microscopy

Cryosections of human ileum biopsy samples were fixed with 4% paraformaldehyde for 15 min, permeabilized with 0.1% Triton X-100 for 20 min at room temperature, and blocked for 1 h at room temperature (BSA 1% in 1X PBS). Slides were then incubated with the rabbit primary antibody against human CD73 (1:100, #12,231–1-AP, Proteintech, Rosemont, Illinois, USA) and mouse primary antibody against human Smad7 (1:50, MAB2029, R&D Systems, Minneapolis, Minnesota, USA) overnight at 4 °C. After washing with 1X PBS, a goat anti-rabbit Alexa 488 (1:2000, #A11008; Invitrogen, Waltham, Massachusetts, USA) or goat anti-mouse Alexa 568 (1:2000, #A11004; Invitrogen) was applied for 1 h at room temperature. Slides were washed with 1X PBS, mounted using Prolong gold antifade reagent with 4′,6-diamidino-2-phenylindole (#P36931; Invitrogen), and analyzed by FLUOVIEW FV4000 confocal laser scanning microscope (Evident Scientific, Tokyo, Japan).

### Immunohistochemistry

Cryosections of murine ileum samples were stained with primary antibodies against mouse Ki67 (1:100, #M7249, Dako, Glostrup, Denmark), mouse p-Smad3 (1:400, #ab84177, Abcam, Cambridge, UK), mouse CD8 (1:100, #251,609, Abcam), mouse CD11c (1:200, #219,799, Abcam), mouse lysozyme (1:500, # ab108508, Abcam) and mouse Lgr5 (1:600, #NBP 1–2-8904, Novus Biologicals). Isotype control-stained sections were prepared under identical immunohistochemical conditions using a rat and rabbit normal IgG control antibody (R&D Systems). Positive cells were visualised using mouse anti-rat/HRP antibody(1:200, #P0450, Dako) and MACH4 Universal HRP-Polymer kit with DAB (Biocare Medical #M4BD534G, Pacheco, California, USA) and analysed by LEICA DMI4000 B microscope with LEICA application suite software (V4.6.2).

### Immunofluorescence staining

Cryosections of ileal samples were placed in methanol–Carnoy’s fixative solution (60% methanol, 30% chloroform, 10% glacial acetic acid) for 30 min at room temperature. Sections were then washed in 1X PBS and permeabilized with 0.1% Triton X-100 for 20 min. Blocking procedure (BSA 1%) was performed for 1 h at room temperature and rabbit primary antibody against mouse mucin-2 (1:100, #sc-15334; Santa Cruz Biotechnology, Dallas, Texas, USA) were incubated overnight at 4° C. After washing with 1X PBS, the secondary antibody goat anti-rabbit Alexa 488 (1:2000, #A11008; Invitrogen) was applied for 1 h at room temperature. Slides were washed with 1X PBS and mounted using Prolong gold antifade reagent with 40,6-diamidino-2-phenylindole (#P36931; Invitrogen) and analyzed by Leica DMI4000 B microscope (Wetzlar, Germany) with Leica application suite software (V4.6.2).

Frozen OCT-embedded murine ileal tissue samples were sectioned at 5 μm thickness for immunofluorescence staining of immune cell markers. Sections were fixed in 4% paraformaldehyde for 10 min, washed, and then blocked for 30 min with 1% BSA in PBS supplemented with 5% normal goat serum (NGS). Tissues were then incubated with primary and secondary antibodies using two distinct staining panels (see Table 1), as follows.

**Table 1:** Primary and secondary antibodies used for the immunofluorescence staining of immune cell markers

Marker	Fluorochrome	Clone	Company	Catalog
CD8	–	CAL38	Abcam	ab251609
CD4	Alexa Fluor488	GK1.5	BioLegend	100,446
CD11c	Alexa Fluor647	N418	BioLegend	117,312
Ly6G	–	EPR22909-135	Abcam	ab238132
B220	Alexa-Fluor594	RA3-6B2	BioLegend	103,254
Goat-αrIgG*	Alexa-Fluor488	–	Thermo Fisher Scientific	A-11008

^*^ goat anti-rabbit IgG (H + L) secondary antibody

In the first panel, sections were incubated overnight at 4 °C with anti-CD8 antibody (1:200), followed by 1-h incubation at RT with Alexa Fluor-488 goat anti-rabbit IgG (1:500). Next, slides were incubated overnight at 4 °C with Alexa Fluor-594 anti-CD4 (2.5ug/ml) and Alexa Fluor-647 anti-CD11c (1:25).

In the second panel, sections were incubated with anti-Ly6G antibody (1:50) overnight at 4 °C, followed by 1-h incubation at RT with Alexa Fluor-488 goat anti-rabbit IgG. Next, slides were incubated overnight at 4 °C with Alexa Fluor-594 anti-B220 antibody (1:100). In separate experiments, sections were incubated with rabbit primary antibody against mouse CD73 (1:100, #12,231–1-AP, Proteintech) overnight at 4 °C. After washing with 1X PBS, a goat anti-rabbit Alexa 488 (1:2000, #A11008; Invitrogen) was applied for 1 h at room temperature.

After staining, slides were counterstained for 5 min with Hoechst (#H3570, Invitrogen) and cover-slipped with 60% glycerol in PBS. Confocal microscopy imaging was performed by Leica TCS-SP8Xlaser-scanning confocal microscope (Leica Microsystems) equipped with a tunable white light laser source, 405 nm diode laser, 3 (PMT) and 2(HyD) internal spectral detector channels. Sequential confocal images were acquired using a HC PLAPO 40 × oil immersion objective (1.30 numerical aperture, Leica Microsystems) with a 1024 × 1024 image format, scan speed 400 Hz. The density of CD8⁺/CD4⁺ T cells, dendritic cells, B cells, and neutrophils infiltrating the tissue was assessed by counting the number of positive cells per unit area (mm^2^). Cell counts were conducted by two independent examiners who were blinded to the samples. For statistical analysis, the average number of positive cells across seven randomly selected fields per sample was calculated.

### Spatial transcriptomics

OCT blocks were cut with a pre-cooled cryostat at 10 μm thickness, and sections were transferred to fit the 6.5 mm^2^ oligo-barcoded capture areas on the Visium 10 × Genomics slide. Visium Spatial Tissue Optimization (10 × Genomics, Pleasanton, California, USA) was performed according to the manufacturer’s instructions, and the fluorescent footprint was imaged using a Metafer Slide Scanning Platform (Metasystems). Thirty minutes was selected as the optimal permeabilization time. The experimental slide with ileal tissue was fixed and stained with hematoxylin and eosin and imaged using a Leica DM5500 B microscope (Leica Microsystems) at 5X magnification. The Leica Application Suite X (LAS X) was used to acquire tile scans of the entire array and merge images. Sequence libraries were then processed according to the manufacturer’s instructions (10 × Genomics, Visium Spatial Transcriptomic). After the second cDNA strand synthesis, cDNA was quantified with quantitative RT-PCR ABI 7500 Fast Real-Time PCR System and analyzed with ABI 7500 Software 2.3.

### Metabolomic analysis

IECs were isolated from ileal samples of Smad7TgCre^+^ and control mice as previously described [30]. Briefly, fresh ileal specimens were cut into 5-mm fragments, and incubated in Dulbecco’s modified Eagle medium containing 15 mmol/L EDTA for 30 min at 4 °C. The supernatants were then centrifuged and IECs were recovered, dried with a SpeedVac (ThermoFisher) and resuspended for LC–MS analysis in 50 µl of 70/30 ACN/H_2_O. Metabolome analyses were performed on a Vanquish Flex UHPLC coupled online to an Exploris 120 hybrid quadrupole Orbitrap mass spectrometer (ThermoFisher Scientific) equipped with a heated electrospray ionization probe (HESIII). Metabolome separation was carried out with a BEHA mide column (100 × 2.1 mm; 1.7 μm) protected with a Vanguard precolumn (5 × 2.1 mm;1.7 μm) (Waters, Milan, Italy). MS1 acquisition was performed at 60.000 resolution, while MS/MS at 15.000 resolution in DDA mode. Detailed LC and MS conditions and pre-processing steps (alignment, filtering, normalization, and annotation). To assess repeatability and instrument stability, a quality control (QC) sample was prepared by pooling 10 µL from each sample. Samples were injected in randomized order, and blank injections were regularly performed to monitor carryover and exclude background signals. After data pre-processing, statistical analyses were conducted using univariate and multivariate approaches (SONDA S.r.l., Salerno, Italy).

### Analysis of 16S rRNA gene amplicon sequencing

Ileal samples were isolated from Smad7TgCre^+^ and control mice, and DNA was extracted (#69,506, Qiagen, Hilden, Germany). 16S rRNA gene amplicon sequencing was performed by IGA Technaology Services (Udine, Italy). QIIME2 was used only to elaborate the relative graphs and statistical analyses, while all the upstream analyses (filtering of the raw data, taxonomic identification and ASV table) were done with the R dada2 package.

### Culture and stimulation of intestinal epithelial cells

IECs isolated from control mice were stimulated with recombinant murine TGF-β1 (20 ng/ml, #7666, R&D Systems, Minneapolis, Minnesota, USA) or adenosine (1 mM, #A4036, Sigma) for 1.5 or 3 h and then  collected and resuspended in lysis buffer supplemented with 1% β-mercaptoethanol and stored at -80 °C until RNA and protein extraction.

### Isolation of lamina propria mononuclear cells and spleen cells.

Lamina propria mononuclear cells (LPMC) were isolated from Smad7TgCre^+^ mice and control mice 4 weeks after the last injection of TMX. Briefly, the dissected murine ileum was freed of mucus and epithelial cells by DTT and EDTA and then digested with DNase (0.1 mg/ml, #11,284,932,001 Roche, Indianapolis, Indiana, USA) and liberase (0.1 mg/ml, #05401127001 Roche) [31]. LPMCs were filtered through a 70-µm cell strainer, spun at 1500 rpm for 5 min, and resuspended in complete RPMI 1640 medium (10% FBS, 1% P/S).

In parallel, spleens were taken from Smad7TgCre^+^ and control mice 4 weeks after the last injection of TMX, cut into small pieces, and gently pressed through a 70-µm cell strainer. Splenocytes were then collected, washed with complete medium, and resuspended in ACK buffer to lyse red blood cells for 1 min. Lysis was then stopped by adding cold medium. Cells were spun at 1500 rpm for 5 min and resuspended in complete RPMI 1640 medium (10% FBS, 1% P/S).

### Flow cytometry

LPMC and splenocytes were stained with the LIVE/DEAD Fixable Dead Cell Stain kit (1:1000, #L34957 Thermo Fisher) for 30 min in ice, washed, and then stained with the following antibodies (all purchased from BD Biosciences, Franklin Lakes, New Jersey, USA): anti-mouse CD45-APCCy7 (#557,659 BD), anti-mouse CD11c-PE (#558,079), anti-mouse Ly6G-APCCy7 (#552,093), anti-mouse CD3-PB (#558,214), anti-mouse CD4-FITC (# 553,729), anti-mouse CD8-PerCP (#551,162), anti-mouse DX5-PE (#553,858), and anti-mouse CD19-FITC (#553,785). The antibody anti-mouse F4/80-PerCP was purchased from eBioscience (#17,480,182, San Diego, California). Appropriate isotype-matched controls were included. The Gallios flow cytometer (Beckman Coulter, Brea, California, USA) was used for the acquisition and the Kaluza software (Beckman Coulter) was used for analysis.

### Western blotting

IECs were lysed on ice in buffer containing 10 mM HEPES (pH 7.9), 10 mM potassium chloride (KCl), 0.1 mM ethylenediaminetetraacetic acid (EDTA), 0.2 mM ethylene glycol-bis (β-aminoethyl ether)-N,N,N′, N′-tetraacetic acid (EGTA), and 0.5% Nonidet P40 supplemented with 1 mM dithiothreitol [DTT], 10 mg/ml aprotinin, 10 mg/ml leupeptin, 1 mM phenylmethylsulphonyl fluoride (PMSF), 1 mM Na3VO4, and 1 mM sodium fluoride (NaF). Lysates were clarified by centrifugation and separated on sodium dodecyl sulphate (SDS) polyacrylamide gel electrophoresis. Blots were incubated with antibodies against Smad7 (1:1000, #37,036, SAB, Greenbelt, Maryland, USA), phosphorylated Smad3 (p-Smad3, 1:1000, #ab84177, Abcam), and β-actin antibody (1:5000, #A544 Sigma), followed by secondary antibodies conjugated to horseradish peroxidase (Dako).

### Real-time PCR

Total RNA was isolated from IECs obtained from the ileal tissues of C57Bl/6 mice or ileal samples from adenosine-treated Smad7TgCre^+^ and control mice using the RNeasy Mini Kit (#74,104 Qiagen, Hilden, Germany) and digested with DNase (Qiagen). A constant amount of RNA (1 μg/sample) was retrotranscribed into complementary DNA (cDNA) using Oligo(dT) primers and M-MLV-reverse transcriptase (#28,025,021 Thermo Fisher Scientific). Real-time PCR was performed for mouse Nt5e (#Mm00501910_m1, Thermo Fisher Scientific), Muc-2, TNF-α, IL-6, IFN-γ, and IL-17A. RNA expression was calculated relative to the β-actin gene using the ΔΔCt algorithm.

### Quantification of fecal Lipocalin-2

Faecal samples were weighted and resuspended in PBS 1X containing 0.1% Tween at a final concentration of 100 mg/ml. Samples were then vortexed for 20 min, centrifuged for 10 min at 14,000 g and 4 °C, and supernatants collected and stored at −80 °C. Lcn-2 protein levels were quantified using the Duoset murine LCN-2 enzyme-linked immunosorbent assay (ELISA) kit (#DY1857, R&D Systems), and optical density was read at 450 nm (DXT880 Multimode detector, Beckman Coulter).

### In vivo intestinal permeability

Intestinal epithelial barrier permeability was assessed using the fluorescein isothiocyanate (FITC)-labeled dextran method. Mice were fasted for 4 h and received 12 mg/mouse of FITC-labeled dextran (4 kDa, #46,944 Sigma) by oral gavage. Blood samples were collected from the submandibular vein (cheek punch) after 1 h and fluorescence intensity was measured in the serum (excitation, 490 nm; emission, 520 nm; DXT880 Multimode detector, Beckman Coulter).

### TUNEL Assay

Cell death was assessed using fluorescence microscopy and the terminal deoxynucleotidyl transferase-mediated dUTP nick-end labeling (TUNEL, #64,936, Cell Signaling) kit. Slides were analyzed by Leica DMI4000 B microscope (Wetzlar, Germany) with Leica application suite software (V4.6.2).

### Statistical analysis

Parametric data were analyzed using the two-tailed Student's t-test for comparison between two groups or one-way ANOVA followed by Tukey's post hoc tests for multiple comparisons. Significance was defined as p < 0.05.

## Results

### Selective overexpression of Smad7 in the mouse intestinal epithelium leads to terminal ileitis

**Fig. 1:**
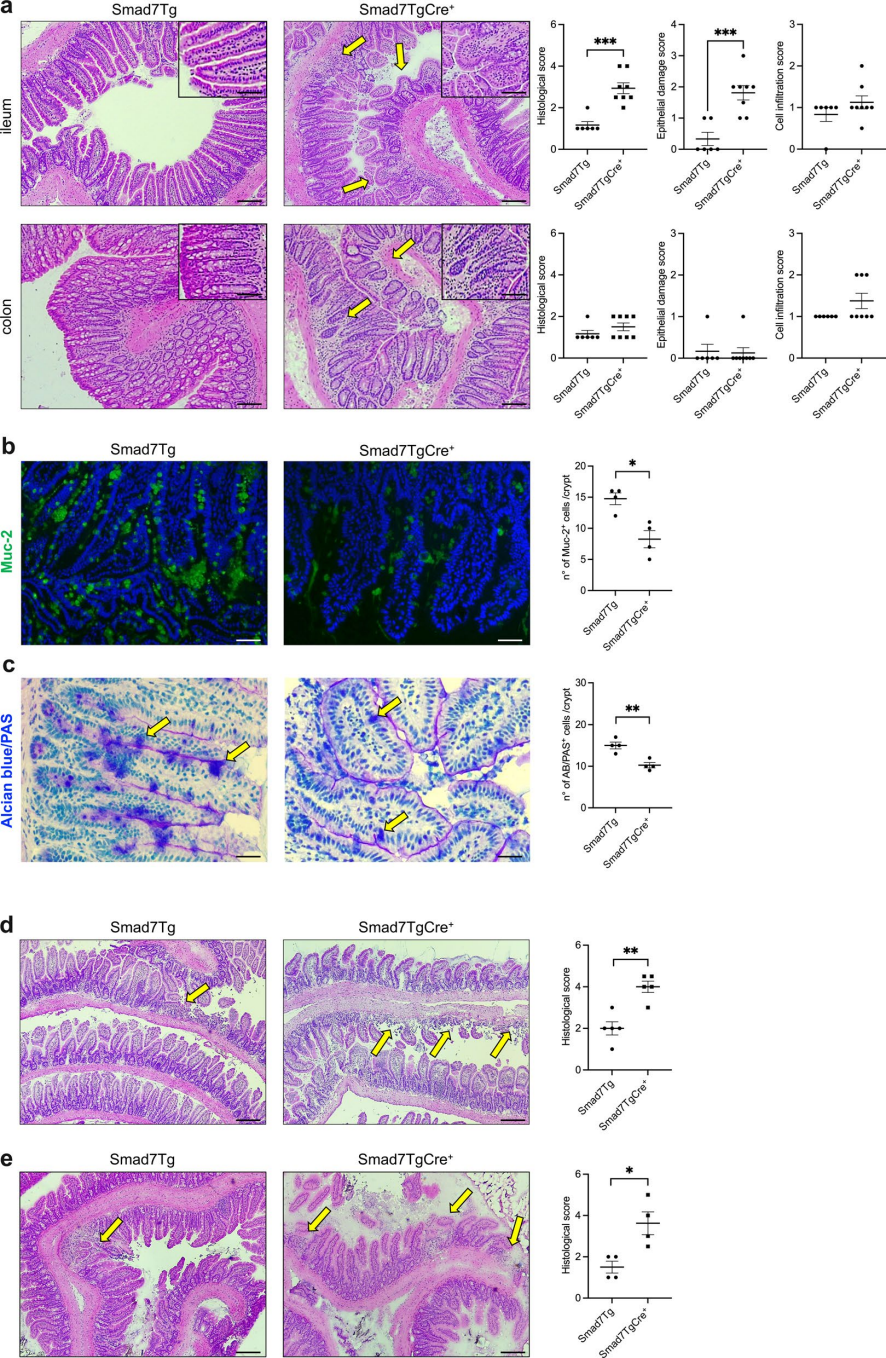
Selective overexpression of Smad7 in the mouse intestinal epithelium leads to terminal ileitis*.* **a**. Representative haematoxylin and eosin (H&E) staining (left panels) and corresponding histological score (right panels) of terminal ileal and colonic sections from Smad7Tg (control) (n = 6) and Smad7TgCre^+^ mice (n = 8) 4 weeks after the last tamoxifen injection. Scale bars: 100 µm. 10 µm (insets). Yellow arrows indicate damaged epithelium. Data in the right graphs are presented as mean ± SEM, with each point representing the total histological score, epithelial damage score, or cell infiltration score for an individual mouse. Differences between groups were analyzed using a two-tailed Student’s *t*-test (*** P ≤ .001). **b, c**. Representative immunofluorescence images and quantification of mucin-2 (Muc-2) (**b**) and Alcian blue/PAS (**c**) positive cells in ileal sections from Smad7Tg (n = 4) and Smad7TgCre^+^ (n = 4) mice treated as indicated in panel **a**. Scale bars: 50 μm. Yellow arrows indicate Alcian blue/PAS-positive cells. Data in the right graph are presented as mean ± SEM, with each point representing the number of positive cells per crypt, counted in four different fields per section. Differences between groups were analyzed using a two-tailed Student’s *t*-test (* P ≤ .05, ** P ≤ .01). **d, e**. Representative H&E staining and histological score of ileal sections from Smad7Tg (n = 4–5) and Smad7TgCre^+^ (n = 4–5) mice treated with indomethacin (5 mg/kg, subcutaneous injection) and sacrificed 24 h later (**d**), or with propyzamide (100 mg/kg, oral gavage) administered every other day and sacrificed after 2 weeks (**e**). Scale bars: 100 μm. Yellow arrows indicate damaged epithelium. Data in the right graph are presented as mean ± SEM, with each point representing the histological score for a single mouse. Differences between groups were analyzed using a two-tailed Student’s *t*-test (* P ≤ .05; ** P ≤ .01)

To assess whether the selective overexpression of Smad7 in the intestinal epithelium drives gut pathology, we generated Smad7Tg VillinCreER^T2+^ (Smad7TgCre^+^) mice by crossing VillinCreER^T2+^ mice with Smad7Tg mice, which carry a green fluorescent protein (GFP)-based reporter system upstream of the Smad7 gene locus (Suppl. Figure 1a). Conditional overexpression of the Smad7 gene in IECs of Smad7TgCre^+^ mice was achieved through intraperitoneal injection of TMX for 5 consecutive days. Western blotting and immunohistochemistry analysis conducted on gut tissue samples harvested 4 weeks after treatment revealed significant overexpression of Smad7 in the IECs isolated from the ileum and colon of Smad7TgCre^+^ mice compared to IECs isolated from control mice (Suppl. Figure 1b), and this was paralleled by decreased levels of phosphorylated Smad3 (p-Smad3) (Suppl. Figure 1c), the latter indicating the impairment of TGF-β1 signaling. However, when analysis was restricted to Smad7TgCre^+^ mice, it was evident that Smad7 expression in ileal IECs was almost 3 times grater than that in colonic samples (Suppl. Figure 1b). Upon dissection, Smad7TgCre^+^ mice had no macroscopic abnormalities, but histological analysis of intestinal Swiss rolls revealed segmental changes in the terminal ileum (Fig. 1a). Specifically, there was a shortening and widening of villi, which appeared as small projections in the lumen of the small intestine. Additionally, we observed focal distortion of the gland architecture, edema, increased inflammatory cell infiltration and epithelial cell death (Suppl. Figure 1d). These changes resulted in a higher histological score of intestinal inflammation compared to the control group (Fig. 1a, right insets). In contrast, in the colon of Smad7TgCre^+^ mice, there were only a focal mucosal infiltration of inflammatory cells and edema, with no morphological changes in the epithelium (Fig. 1a). Smad7TgCre^+^ mice showed also a marked reduction of Mucin-2 (Muc-2)-positive cells in ileal samples of Smad7TgCre^+^ compared to the controls (Fig. 1b), which was consistent with the Alcian Blue-Periodic Acid-Schiff (AB-PAS) staining for goblet cells (Fig. 1c). In contrast, there was no significant change in Ki-67-positive cell staining between the two groups (Suppl. Figure 1e).

Next, we assessed whether epithelial overexpression of Smad7 exacerbates experimental gut pathology induced by compounds that are supposed to contribute to IBD development, namely nonsteroidal anti-inflammatory drugs and pesticides [32, 33]. As compared to controls, Smad7TgCre^+^ mice developed a more severe ileitis when challenged with a single subcutaneous injection of indomethacin (Fig. 1d). Similarly, Smad7 epithelial overexpression exacerbated the gut pathology induced by oral administration of propyzamide (Fig. 1e), a common herbicide that is known to upregulate NF-κB-driven C/EBPβ inflammatory genes and inhibit AHR signaling pathways in the gut [33].

Altogether, these data indicate that selective overexpression of Smad7 in IECs elicits signals that culminate in the development of ileal damage.

### *Smad7-overexpressing mice exhibit enhanced mucosal CD8*^+^*T cell infiltration*

**Fig. 2:**
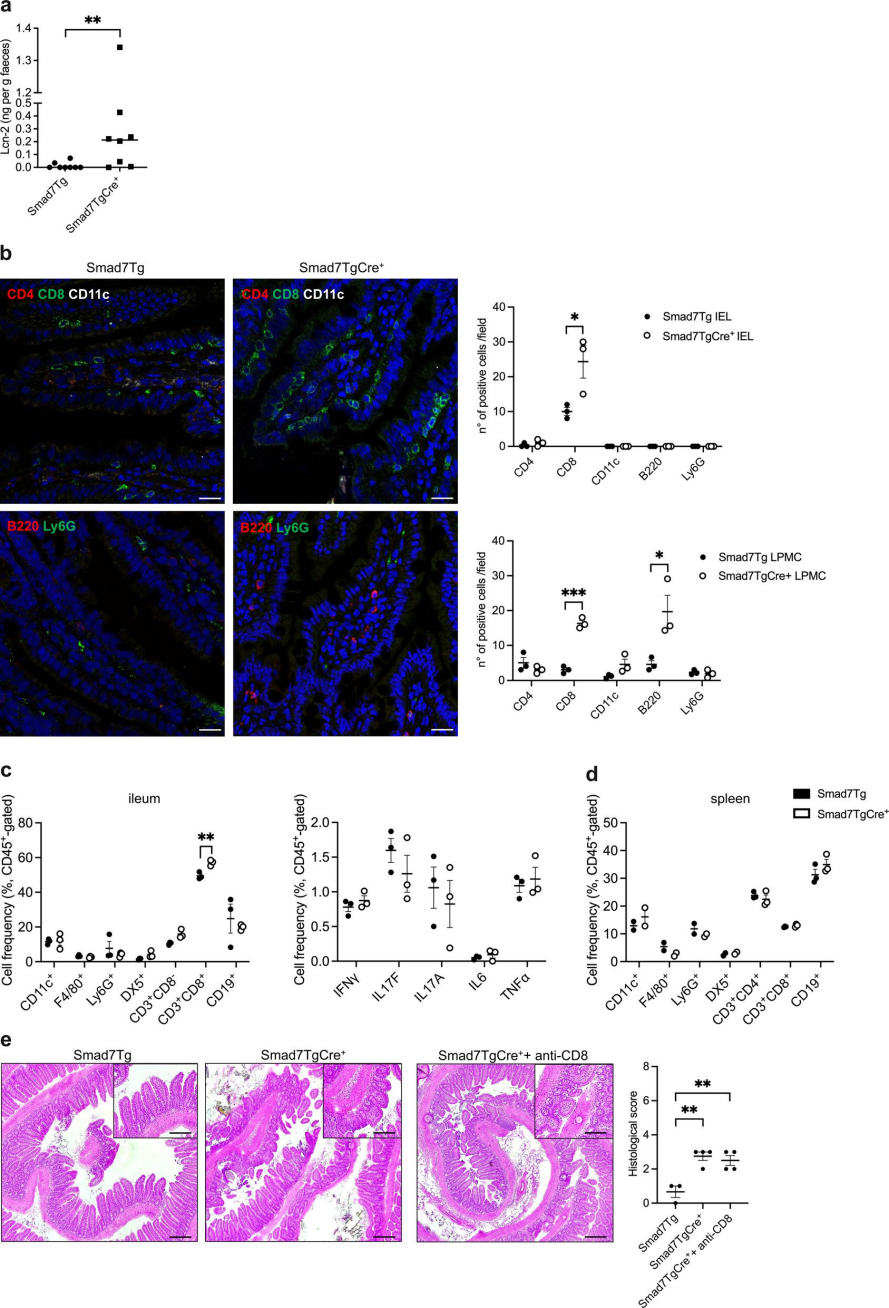
Smad7TgCre^+^ mice exhibit enhanced CD8^+^ T cell mucosal infiltration. **a**. Dot plot showing levels of fecal lipocalin-2 (Lcn-2) protein in Smad7Tg (controls) (n = 8) and Smad7TgCre^+^ mice (n = 8) 4 weeks after the last tamoxifen injection. Each point corresponds to the level of Lcn-2 protein in the faeces of a single mouse; horizontal bar indicates the median value. Differences between groups were analyzed using the Mann–Whitney U test (** P ≤ .01). **b**. Immunofluorescence pictures showing CD4^+^ (red), CD8^+^ (green) and CD11c^+^ (white) cells (upper row), and B220^+^ (red) and Ly6G^+^ (green) cells (lower row) in ileal tissue samples from Smad7Tg (n = 3) and Smad7TgCre^+^ mice (n = 3) treated as described in panel **a**. Scale bars: 25 μm. Right insets: number of CD4-, CD8-, CD11c-, B220-, and Ly6G-expressing cells among intraepithelial lymphocytes (IEL) and lamina propria mononuclear cells (LPMC) of ileal tissue samples taken from Smad7Tg (n = 3) and Smad7TgCre^+^ mice (n = 3) treated as described in panel **a**. Data indicate the mean ± SEM of the positive cells in seven representative selected fields per sample. Differences between groups were analyzed using a two-tailed Student’s *t*-test (* P ≤ .05). **c**. Left panel: dot plot showing the percentages of dendritic cells (CD45^+^CD11c^+^), macrophages (CD45^+^F4/80^+^), neutrophils (CD45^+^Ly6G^+^), NK cells (CD45^+^DX5^+^), CD4 T cells (CD45^+^CD3^+^CD8^−^), CD8 T cells (CD45^+^CD3^+^CD8^+^) and B cells (CD45^+^CD19^+^) in the lamina propria of ileal tissue samples taken from Smad7Tg and Smad7TgCre^+^ mice treated as in described in panel **a**. Data indicate mean ± SEM and were generated by analyzing 3 pools of LPMCs isolated from 2 mice/pool. Differences between groups were analyzed using a two-tailed Student’s *t*-test (** P ≤ .01). Right panel: dot plot showing the fractions of cells producing the indicated cytokines in the ileal lamina propria of Smad7Tg and Smad7TgCre^+^ mice treated as indicated in panel **a**. Data indicate mean ± SEM and were generated by analyzing 3 pools of LPMCs isolated from 2 mice/pool. **d**. Dot plot showing the percentages of dendritic cells (CD45^+^CD11c^+^), macrophages (CD45F4/80^+^), neutrophils (CD45^+^Ly6G^+^), NK cells (CD45^+^DX5^+^), CD4^+^ T cells (CD45^+^CD3^+^CD8^−^), CD8^+^ T cells (CD45^+^CD3^+^CD8^+^) and B cells (CD45^+^CD19^+^) in the spleens taken from Smad7Tg (n = 4) and Smad7TgCre^+^ mice (n = 4) treated as described in panel **a**. Data indicate mean ± SEM. **e**. Representative haematoxylin and eosin staining (left panel) and corresponding histological score (right panel) of distal ileal tissue sections from Smad7Tg (n = 3), Smad7TgCre^+^ (n = 4), and Smad7TgCre^+^ mice treated with intraperitoneal injections of anti-mouse CD8 antibody (5 mg/kg, n = 4) administered 4 times, starting the day after the last TMX injection and then every 3 days. Scale bars: 100 µm. 10 µm (insets). Data in the right graph are presented as mean ± SEM, with each point representing the histological score for a single mouse. Differences among groups were analyzed using one-way analysis of variance (ANOVA) followed by the Tukey test (** P ≤ .01)

In light of the histological analysis indicating an increased inflammatory cell infiltration of the lamina propria of Smad7TgCre^+^ mice, we performed a characterization of the mucosal inflammation in such mice. Initially, we documented higher levels of fecal lipocalin 2 (Lcn-2), a sensitive marker of inflammation, in Smad7TgCre^+^ mice than in controls (Fig. 2a). Analysis of the inflammatory cell infiltration by multiplex immunofluorescence revealed an increased frequency of CD8^+^ T lymphocytes in the ileal samples of Smad7TgCre^+^ mice compared to controls (Fig. 2b), while no significant changes were seen in terms of CD4^+^ T lymphocytes, dendritic cells (CD11c^+^), and neutrophils (Ly6G^+^) (Fig. 2b). Moreover, ileal sections of Smad7TgCre^+^ mice had a focal increase of B cells (B220^+^) in the lamina propria as compared to controls (Fig. 2b).

To corroborate these data, we isolated LPMC from ileal samples of Smad7TgCre^+^ mice and control mice and evaluated the phenotype of the CD45^+^ LPMC and the percentages of cytokine-expressing LPMC by flow cytometry. CD45^+^ LPMC isolated from ileal samples of Smad7TgCre^+^ mice exhibit only a significant increase in CD3^+^CD8^+^ T lymphocytes as compared to controls (Fig. 2c, left panel), while there was no significant change in the frequency of cytokine-expressing CD45^+^ LPMC (Fig. 2c, right panel). To assess whether the increase in CD3^+^CD8^+^ T lymphocytes in the ileal LPMC preparations of Smad7TgCre^+^ mice reflected either a local or systemic immune response, we assessed the phenotype of CD45^+^ splenocytes isolated from Smad7TgCre^+^ mice and control mice. No significant change in the fractions of CD45^+^ splenocytes was seen between the two groups of mice (Fig. 2d).

To test whether the ileal morphological changes seen in Smad7TgCre^+^ mice were due to the cytotoxic action of CD3^+^CD8^+^ T lymphocytes, we monitored such alterations in mice treated with a depleting CD8^+^ antibody. Depletion of more than 85% of CD3^+^CD8^+^ T cells did not prevent the intestinal epithelial alterations (Fig. 2e, Suppl. Figure 2), arguing against a role for CD8^+^ T cells in the Smad7-induced epithelial damage.

### Smad7-overexpressing mice exhibit mucosal dysbiosis

**Fig. 3:**
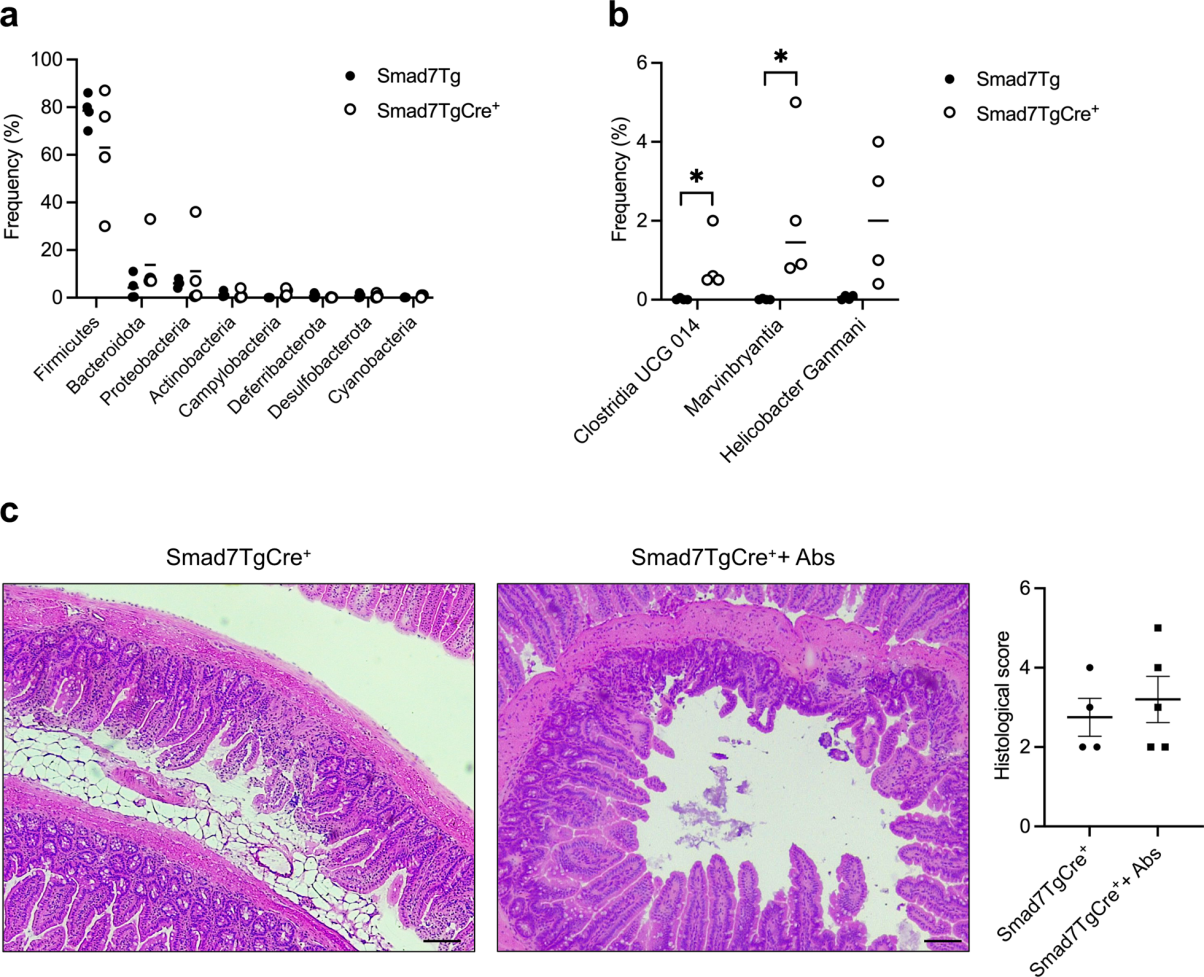
Smad7-overexpressing mice exhibit mucosal dysbiosis. **a, b.** Relative abundance of phyla (**a**) and selected orders and genera (**b**) of ileal mucosa-associated microbiota in Smad7Tg (n = 4), and Smad7TgCre^+^ mice (n = 4) 4 weeks after the last tamoxifen injection. Data are expressed as median values, and each point corresponds to a single mouse. Differences between groups were analyzed using the Mann–Whitney U test (* P ≤ .05). **c**. Representative haematoxylin and eosin staining (left panel) and histological score (right panel) of ileal sections taken from Smad7TgCre^+^ mice treated (n = 5) or untreated (n = 4) with antibiotics for 2 weeks, starting on day 14 after the last tamoxifen administration. Scale bars: 100 µm. Data in the right graph are presented as mean ± SEM, with each point representing the histological score of a single mouse

To determine whether the selective over-expression of Smad7 in epithelial cells impacts the composition of gut microbiota, the bacterial communities from ileal samples derived from Smad7TgCre^+^ mice and wild-type mice were analyzed by 16S rRNA sequencing. Selective over-expression of Smad7 in IECs was associated with no major bacterial changes in terms of phylum (Fig. 3a), but there were some alterations in terms of order and genus (Fig. 3b). In particular, Smad7TgCre^+^ mice had a significant increase in the frequency of *Clostridia UCG 014* and *Marvinbryantia*, both belonging to the phylum *Firmicutes*, and increased, but not significant levels of *Helicobacter Ganmani*(Fig. 3b). To better understand the role of microbiota in the intestinal morphological changes seen in Smad7TgCre^+^ mice, we depleted gut microbiota with a broad-spectrum antibiotic cocktail in drinking water for 2 weeks. The antibiotic cocktail was started 14 days after the last injection with TMX, because at this time point, the Smad7TgCre^+^ mice did not exhibit major morphological changes. Antibiotic treatment did not prevent/reverse the main epithelial alterations (Fig. 3c).

### Smad7 overexpression in ileal epithelial cells impairs purine metabolism

**Fig. 4:**
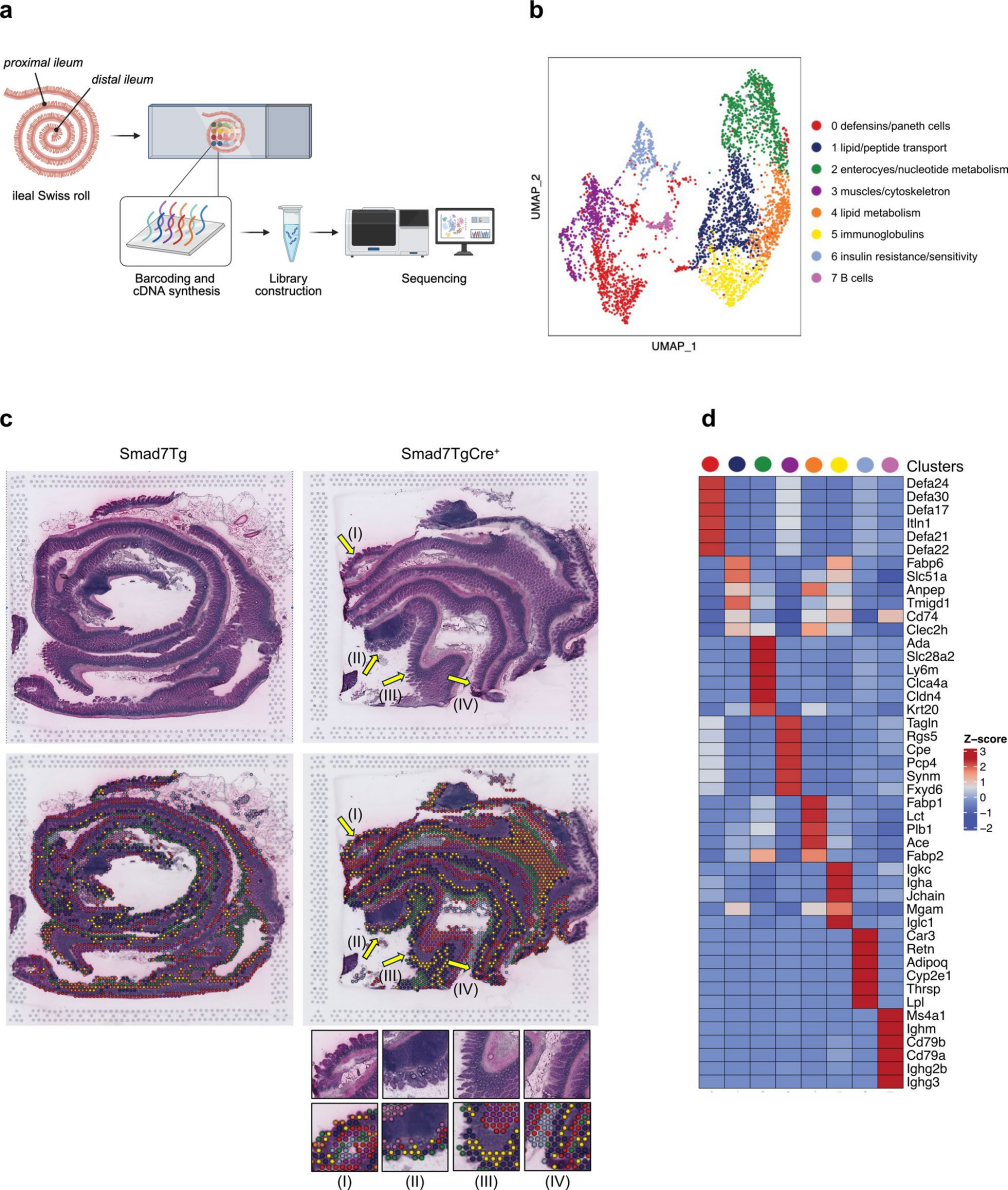
**Genes are differentially expressed in different clusters. a.** Schematic representation of spatial transcriptomic analysis: ileal tissues from Smad7Tg mice (n = 1) and Smad7TgCre^+^ mice (n = 1) were isolated 4 weeks after the last tamoxifen injection and processed as a Swiss roll for spatial transcriptomic (ST) with Visium 10X technology. **b**. Uniform Manifold Approximation and Projection (UMAP) representation of 8 color-coded clusters defining regional transcriptome diversity in the Smad7Tg and Smad7TgCre^+^ mice datasets combined. **c**. Ileal Swiss rolls stained with hematoxylin and eosin (top). Yellow arrows indicate areas of damaged epithelium. Magnifications of the damaged epithelium areas of the ileal Swiss roll from Smad7TgCre^+^ mice showing the distribution of the color-coded clusters (bottom). **d**. Heatmap showing the top genes defining each color-coded cluster in the ST datasets

**Fig. 5:**
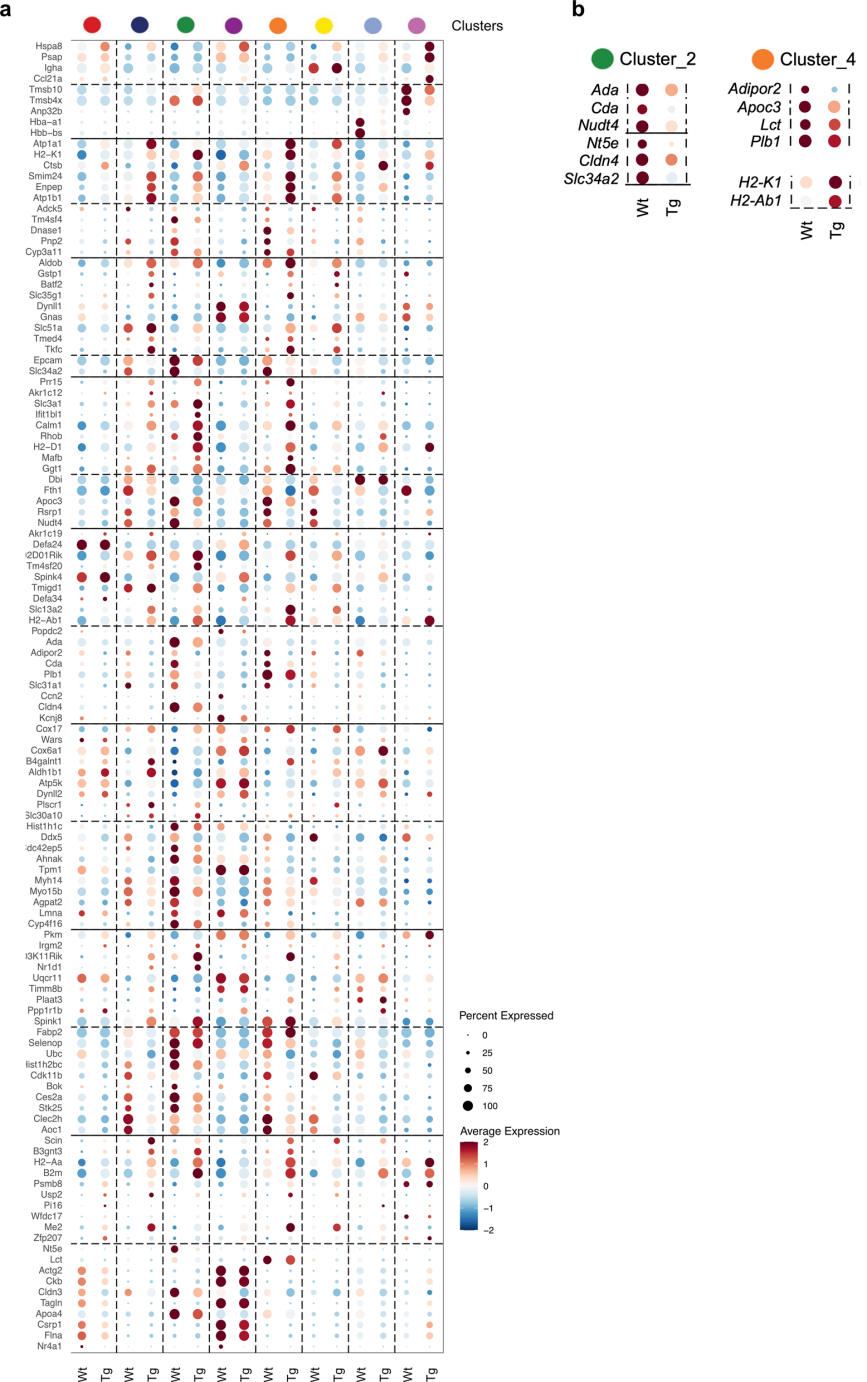
Genes are differentially expressed across the various group clusters.** a.** Heatmap showing the top genes defining each color-coded cluster in the spatial transcriptomic datasets from the ileal samples taken from Smad7Tg (Wt, n = 1) and Smad7TgCre^+^ (Tg, n = 1) mice 4 weeks after the last tamoxifen injection. **b**. Heatmap showing the top DEGs in clusters 2 and 4 in the spatial transcriptomic datasets from Smad7Tg (Wt) and Smad7TgCre^+ ^(Tg) mice

**Fig. 6:**
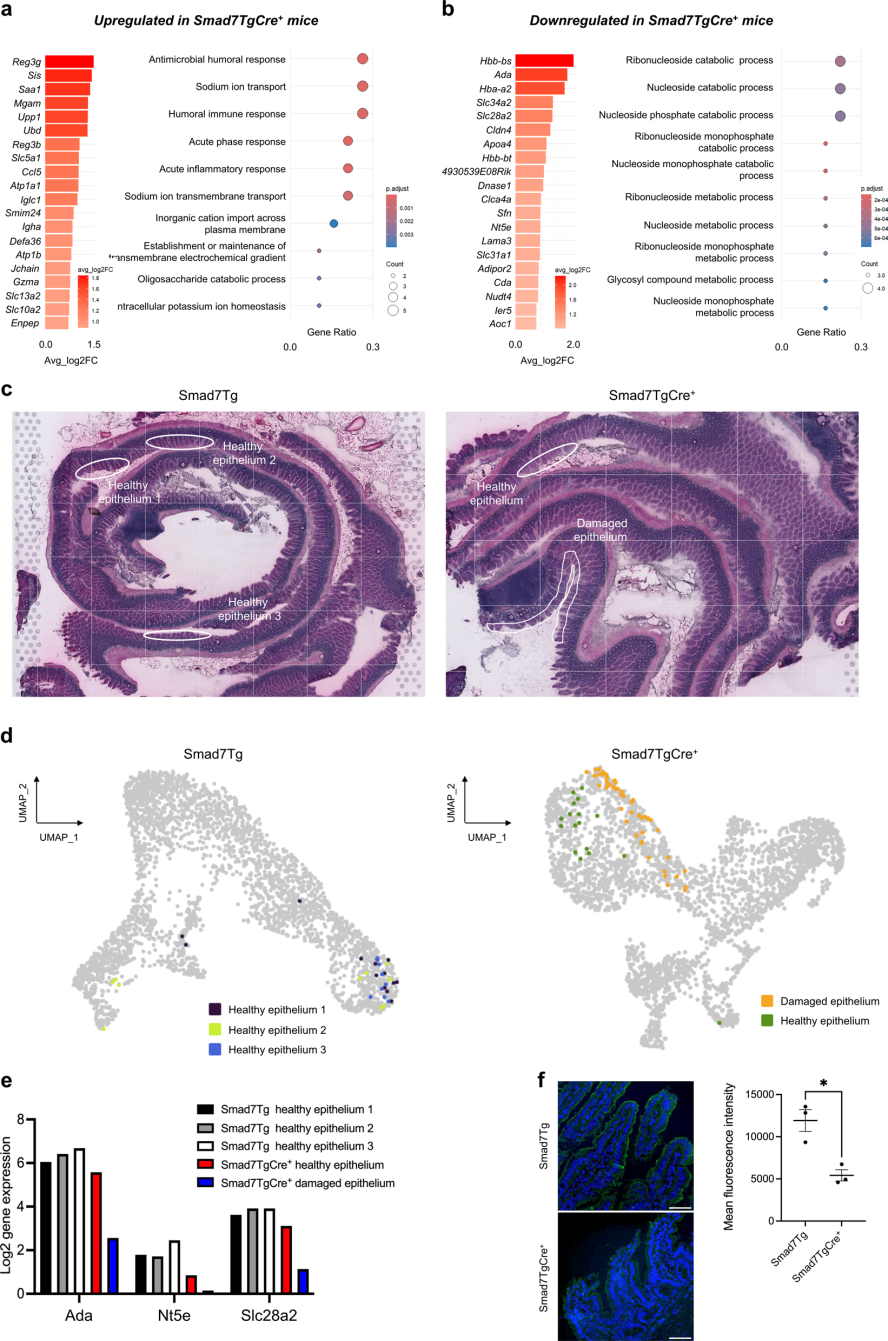
Smad7TgCre^+^ mice exhibit a marked upregulation of anti-microbial and inflammatory responses and downregulation of purine metabolism. **a**. Barplot showing the top 20 upregulated genes ranked by average log2 fold change (avg_log2FC), calculated on the mean gene expression in Smad7tgCre^+^ mice (n = 1) compared to controls (n = 1) 4 weeks after the last tamoxifen injection (left panel). Gene Ontology (GO) biological process enrichment analysis was performed on the same set of 20 upregulated genes (right panel). **b**. Barplot showing the top 20 downregulated genes ranked by average log2 fold change (avg_log2FC), calculated on the mean gene expression in Smad7tgCre^+^ mice (n = 1) compared to controls (n = 1) (left panel). Gene Ontology (GO) biological process enrichment analysis was performed on the same set of 20 downregulated genes (right panel). **c.** Representative epithelial regions selected for gene expression analysis. In Smad7Tg mice (left panel), three distinct areas of healthy epithelium were selected (epithelium-1, violet; epithelium-2, green; epithelium-3, blue). In Smad7TgCre^+^ mice (right panel), regions of healthy epithelium (orange) and damaged epithelium (dark green) were selected to assess *Ada*, *Nt5e*, and *Slc28a2* gene expression. **d.** Uniform Manifold Approximation and Projection (UMAP) representation of the transcriptomic profiles of the epithelial regions indicated in panel **c**, illustrating regional transcriptome diversity among healthy epithelium-1 (violet), -2 (green), and -3 (blue) in Smad7Tg mice (left panel) and healthy (orange) and damaged epithelium (dark green) in Smad7TgCre^+^ mice (right panel). **e**. Histograms showing mean values of *Ada*, *Nt5e*, and *Slc28a2* log2 gene expression in the selected areas. **f.** Left panel: representative immunofluorescence images showing CD73-positive cells in ileal tissue samples from Smad7Tg (n = 3) and Smad7TgCre^+^ mice (n = 3) treated as indicated in panel **a**. Scale bars: 50 μm. **f. **The right panel shows the mean fluorescence intensity (MFI) for CD73. Data are presented as mean ± SEM, with each point representing the MFI in a single mouse. Differences between groups were analyzed using a two-tailed Student’s *t*-test (* P ≤ .05)

To investigate the molecular mechanisms underlying ileal mucosal damage in Smad7TgCre^+^ mice, we performed spatial transcriptomic analysis on frozen ileal samples from both control and Smad7TgCre^+^ mice using the Visium platform (10X Genomics) (Fig. 4a). The pre-filtered dataset consisted mostly of protein-coding genes (Suppl. Figure 3a). After excluding non-coding RNAs and mitochondrial protein-coding genes, we conducted cluster analysis on the remaining dataset. We annotated 8 distinct clusters (Fig. 4b), each associated with unique molecular functions or cell types. Most of them were evenly distributed across the entire ileum of both control and SmadTgCre^+^ mice (Fig. 4c), with some exceptions. For instance, cluster 2 showed reduced expression in regions of Smad7TgCre^+^ mice with damaged epithelium (Fig. 4c, yellow arrows). Differentially upregulated genes for each cluster are summarized in a heatmap (Fig. 4d). Among these clusters, we identified the top genes defining each cluster in control and Smad7TgCre^+^ mice to assess the characteristics of each cluster in each group (Fig. 5a). Notably, cluster 2, which is associated with enterocytes and nucleotide metabolism, and cluster 4, linked to lipid metabolism, displayed the highest number of differentially expressed genes (DEGs) between the two groups of mice (Fig. 5a). Specifically, several enterocyte-related genes (*Cldn4, Slc34a2*) and nucleotide metabolism-related genes (*Ada, Cda, Nt5e, and Nudt4*) in cluster 2 were downregulated in Smad7TgCre^+^ mice compared to controls (Fig. 5b). Additionally, several genes from cluster 4 related to lipid metabolism (*Adipor2, Apoc3, Lct, and Plb1*) were downregulated, while genes involved in immune responses (*H2-K1, H2-Ab1*) were upregulated in Smad7TgCre^+^ mice compared to controls (Fig. 5b). This suggests that clusters 2 and 4 are key discriminators between the two groups. We then identified the most significantly upregulated (Fig. 6a) and downregulated (Fig. 6b) differentially expressed genes (DEGs) in Smad7TgCre^+^ mice compared to controls, irrespective of cluster identity. A total of 143 genes were differentially expressed in the ileum of Smad7TgCre^+^ mice compared to controls, with 67 genes upregulated and 76 downregulated in Smad7TgCre^+^ mice. Functional enrichment analysis using Gene Ontology (GO) revealed that the top 20 upregulated genes in Smad7TgCre^+^ mice were primarily associated with antimicrobial responses (e.g., *Reg3β, Reg3γ*) and acute-phase responses (e.g., *Saa1*) (Fig. 6a), further supporting the inflammatory state in these animals. Among the top 20 downregulated genes, many were linked to ribonucleoside and purine metabolism pathways (Fig. 6b). In particular, Smad7TgCre^+^ mice exhibited a marked downregulation of the *Ada* gene that encodes for adenosine deaminase, which catalyzes the conversion of adenosine to inosine in the purine catabolic pathway. Moreover, the *Slc28a2* gene, that encodes for the solute carrier family 28 member, involved in neurotransmitter, nucleoside, and purine nucleobase transmembrane transport, was also downregulated, along with the *Nt5e* gene, encoding Ecto-5’-nucleotidase/CD73, the rate-limiting enzyme in adenosine production, which serves as the substrate for adenosine deaminase activity [34]. Spatial transcriptomic analysis of selected healthy epithelium in control mice (epithelium-1, -2 and -3), and both healthy and damaged epithelium in Smad7TgCre^+^ mice (Fig. 6c, d, Suppl. Figure 3b) further confirmed the impairment in purine nucleotide metabolism in the damaged epithelium of Smad7TgCre^+^ mice (Fig. 6e). In particular, the expression of *Ada*, *Nt5e*, and *Slc28a2* genes was lower in the damaged epithelium of Smad7TgCre^+^ mice compared to the healthy epithelium of both Smad7Tg and Smad7TgCre^+^ mice. (Fig. 6e). Decreased CD73 protein expression (encoded by *N5te* gene) was also seen in ileal samples from Smad7TgCre^+^ mice compared to controls (Fig. 6f).

**Fig. 7:**
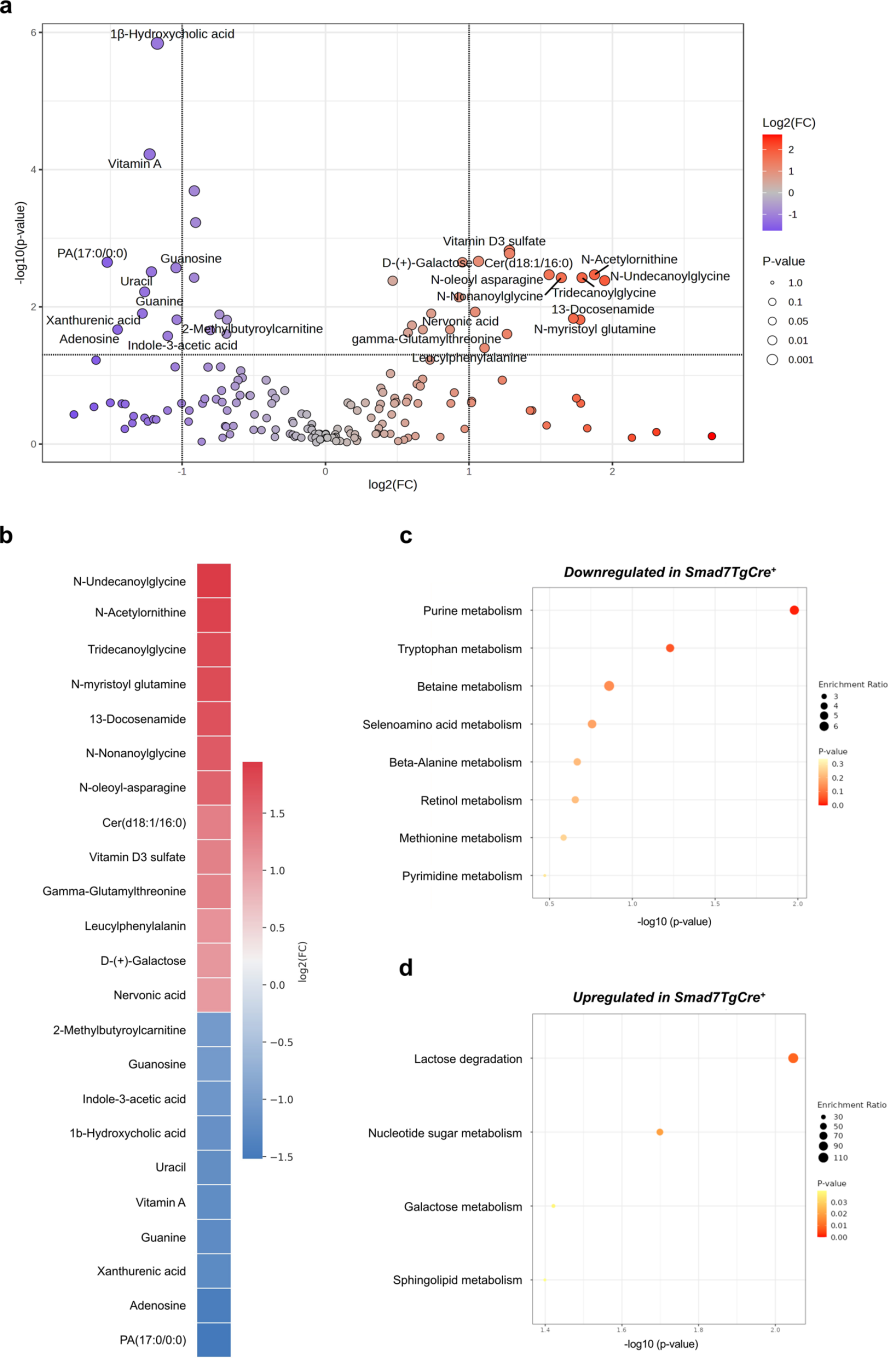
Intestinal epithelial cells of Smad7TgCre^+^ mice exhibit decreased levels of adenosine, guanine, and guanosine metabolites.** a.** Volcano plot of metabolites quantified in the ileal IECs isolated from Smad7tgCre^+^ mice (n = 5) compared to controls (n = 4) 4 weeks after the last tamoxifen injection, displaying log2 fold change (log2FC) on the x-axis and negative log10-transformed p-values (–log10(p-value)) on the y-axis. **b.** Heatmap showing the top differentially abundant metabolites identified in panel **a,** ranked by log2 fold change (log2FC). Results were obtained from the comparison between Smad7tgCre^+^ mice and controls. **c-d**. Metabolic pathway enrichment analysis performed using MetaboAnalyst on the set of significantly downregulated (**c**) or upregulated (**d**) metabolites identified in panel **a**

To validate these findings, we performed a metabolomic profiling of IECs isolated from fresh ileal samples of Smad7TgCre^+^ mice and controls. Twenty-three metabolites were differentially present between the two groups, with 10 metabolites decreased and 13 increased in Smad7TgCre^+^ mice compared to controls (Fig. 7a, b). In line with the above data, purine metabolism was identified as the most significantly impaired pathway in the IECs of Smad7TgCre^+^ mice, as evidenced by the reduction of adenosine, guanine, and guanosine (Fig. 7c). Moreover, IECs of Smad7TgCre^+^ mice exhibited upregulation of the lactose degradation pathway (Fig. 7d).

These data indicate that overexpression of Smad7 in IECs influences cellular metabolism by reducing purine levels and increasing energy demand.

### TGF-β1 enhances Nt5e expression in ileal epithelial cells

**Fig. 8:**
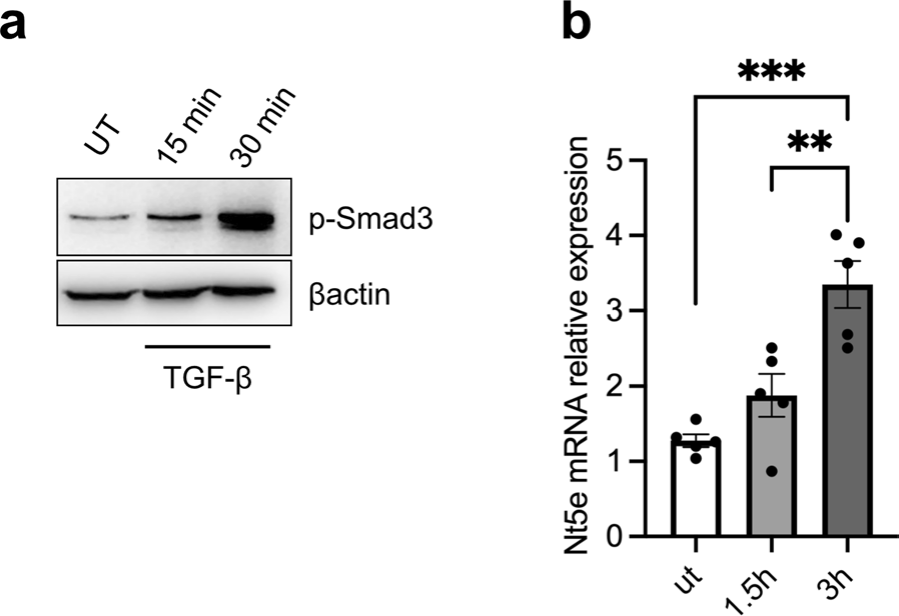
TGF-β1 enhances CD73 expression in ileal epithelial cells.** a**. Representative Western blots of phosphorylated Smad3 (p-Smad3) and β-actin in IECs isolated from the ileum of Smad7Tg mice (n = 1) and stimulated or not with recombinant murine TGF-β1 (20 ng/ml) for 15 and 30 min. **b.** Expression of *Nt5e* RNA transcripts in intestinal epithelial cells isolated from the ileum of Smad7Tg mice (n = 5) and stimulated or not with recombinant murine TGF-β1 (20 ng/ml) for 1.5 and 3 h. Data are presented as mean ± SEM, with each point representing the expression of *Nt5e* in a single sample. Differences among groups were compared using one-way analysis of variance (ANOVA) followed by the Tukey test (** P ≤ .01, *** P ≤ .001). Ut: untreated

Since *Nt5e* was one of the most upstream genes in purine metabolism to be significantly reduced in Smad7TgCre^+^ mice, and previous studies in rat liver myofibroblasts identified one SMAD binding site on *Nt5e* promoter [35], we hypothesized that the downregulation of *Nt5e* transcripts in Smad7TgCre^+^ mice could rely on the Smad7-mediated inhibition of TGF-β1 signaling. To test this hypothesis, primary IECs were isolated from ileal samples of control mice, stimulated in vitro with recombinant TGF-β1, for 1.5–3 h, and then p-Smad3 and *Nt5e* were evaluated by Western blotting and real-time PCR, respectively. TGF-β1 significantly enhanced p-Smad3 and *Nt5e* RNA transcripts (Fig. 8a, b).

### Adenosine administration to Smad7TgCre^+^ mice attenuates ileal mucosal damage

**Fig. 9:**
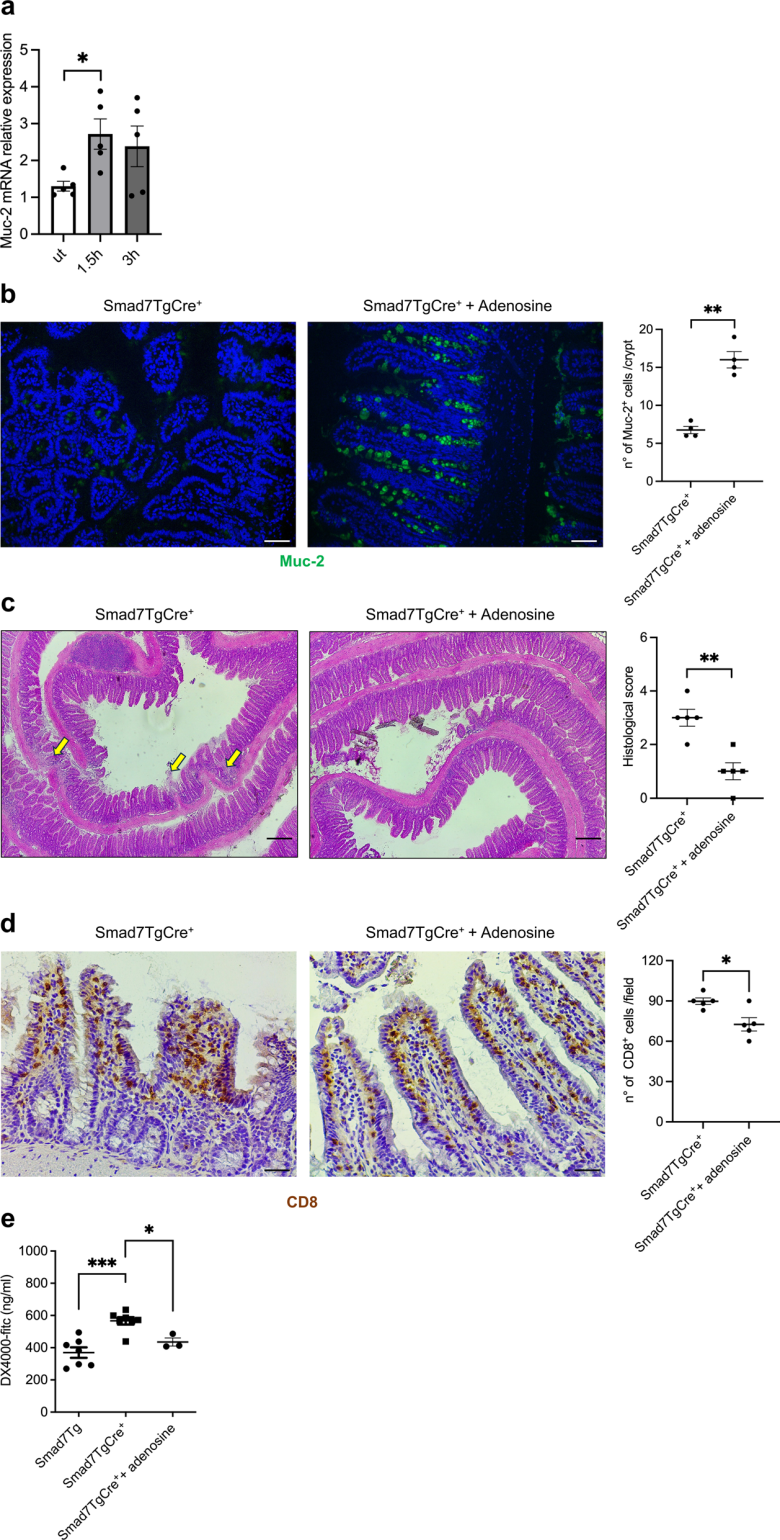
Adenosine administration to Smad7TgCre^+^ mice attenuates ileal damage. **a.** Expression of *Muc-2* RNA transcripts in intestinal epithelial cells isolated from the ileum of Smad7Tg mice (n = 5) stimulated or not with recombinant murine adenosine (1 mM) for 1.5 and 3 h. Data in the right graph are presented as mean ± SEM, with each point representing the percentage of positive cells in a single sample. Differences among groups were compared using one-way analysis of variance (ANOVA) followed by the Tukey test (* P ≤ .05). Ut: untreated. **b**. Representative immunofluorescence images and count of mucin-2 (Muc-2) positive cells in the ileal sections from Smad7TgCre^+^ mice treated with adenosine (10 mg/Kg, n = 4) or vehicle (n = 4) by oral gavage every other day starting the day after the last treatment with tamoxifen until day 28. Data in the right graph are presented as mean ± SEM, with each point representing the number of positive cells per crypt, counted in four different fields per section. Differences between groups were analyzed using a two-tailed Student’s *t*-test (** P ≤ .01). Scale bar: 50 µm. **c**. Representative haematoxylin and eosin staining (left panel) and histological score (right panel) of ileal sections taken from Smad7TgCre^+^ mice treated (n = 5) or untreated (n = 5) as in **b**. Data in the right graph are presented as mean ± SEM, with each point representing the histological score for a single mouse. Differences between groups were analyzed using a two-tailed Student’s *t*-test (** P ≤ .01). Scale bars: 100 µm. **d.** Representative immunohistochemistry images showing CD8-positive cells in the ileal sections from Smad7TgCre^+^ mice treated with adenosine (10 mg/Kg, n = 4) or vehicle (n = 4) by oral gavage every other day starting the day after the last treatment with tamoxifen until day 28. Data in the right graph are presented as mean ± SEM, with each point representing the number of positive cells counted in four different fields per section. Differences between groups were analyzed using a two-tailed Student’s t-test (** P ≤ .01). Scale bar: 50 µm. **e.** Levels of serum FITC-dextran in Smad7Tg (n = 7) and Smad7TgCre^+^ mice, the latter receiving either oral adenosine (10 mg/Kg, n = 3) or vehicle (n = 7). Data in the graph are presented as mean ± SEM with each point representing serum FITC-dextran concentration in a single mouse. Differences among groups were analyzed using one-way analysis of variance (ANOVA) followed by the Tukey test (* P ≤ .05; *** P ≤ .001)

Adenosine is a key regulator of intestinal epithelial barrier integrity and repair [36]. Therefore, we explored the possibility that the ileal mucosal damage seen in Smad7TgCre^+^ mice could be secondary to the reduced production of adenosine. Since the major epithelial defect seen in Smad7TgCre^+^ mice was the reduced production of mucin, we initially assessed the regulatory effects of adenosine on *Muc-2* expression in primary IECs isolated from control mice. Adenosine treatment increased *Muc-2* RNA transcripts (Fig. 9a). Next, we treated Smad7TgCre^+^ mice with oral adenosine or vehicle (control) every other day for 3 weeks, starting from the day after the last treatment with TMX (Suppl. Figure 4). Adenosine-treated mice exhibited a marked up-regulation of Muc-2 protein as compared to control mice (Fig. 9b) and this was paralleled by a significant reduction of the ileal mucosal damage, as focal lesions, villi distortions, and immune cell infiltration were significantly reduced (Fig. 9c). Additionally, adenosine-treated mice displayed a reduced frequency of CD8⁺ T cells (Fig. 9d). Consistent with these findings, intestinal permeability, which was increased in Smad7TgCre⁺ mice compared to controls, was restored by adenosine supplementation (Fig. 9e). In contrast, adenosine treatment did not affect the fractions of Paneth cells (Lyz), stem cells (Lgr5), and proliferating cells (Ki67) (Suppl. Figure 5a-c). Finally, no significant change in the RNA expression of IL-6, TNF-α, IFN-γ, and IL-17A was seen following adenosine treatment (Suppl. Figure 6).

### In the inflamed ileum of CD patients, increased Smad7 correlates with reduced CD73 expression

**Fig. 10:**
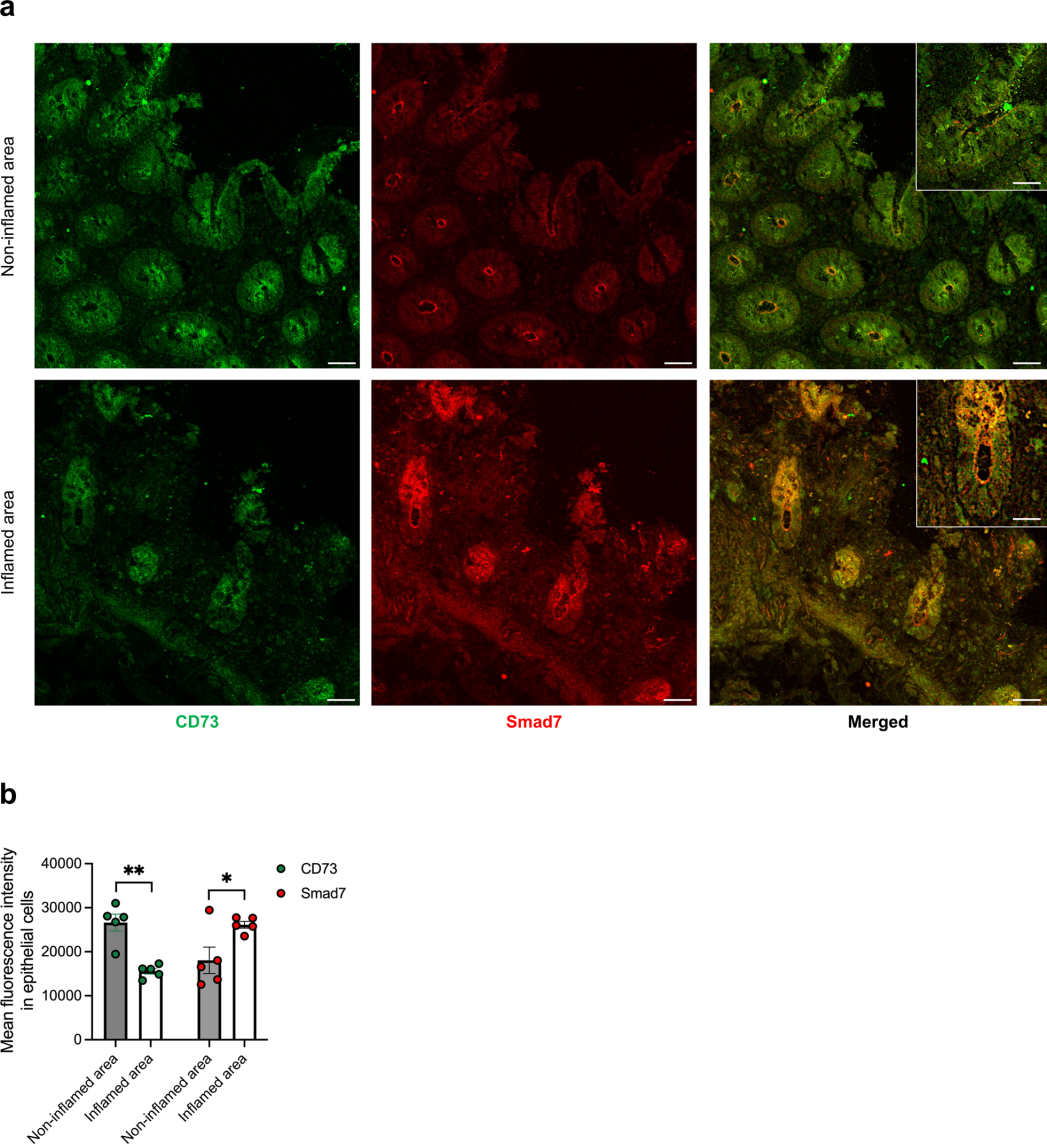
In the epithelial compartment of the inflamed ileum of CD patients, the increased expression of Smad7 associates with a reduction in CD73 expression.** a**. Representative confocal microscopy images of non-inflamed and inflamed ileal sections from CD patients (n = 5) showing CD73 (green) and Smad7 (red) positive cells. Scale bar: 50 µm. 5 µm (insert). **b.** Bar graph showing the mean fluorescence intensity for CD73 and Smad7 in epithelial cells of intestinal sections taken from non-inflamed (grey bar) and inflamed (white bar) tissues of CD patients (n = 5) in four representative selected fields per sample. Data are presented as mean ± SEM, with each point representing the MFI in a single patient. Differences between groups were analyzed using a two-tailed Student’s *t*-test (* P ≤ .05, ** P ≤ .01)

In a final set of experiments, we examined CD73 and Smad7 protein expression in CD tissue. Confocal analysis of ileal mucosal samples from both inflamed and non-inflamed areas showed that, as previously reported [25], Smad7 expression was higher in the epithelial compartment of inflamed areas (Fig. 10a, b). In contrast, CD73 fluorescence intensity was reduced in epithelial cells from inflamed regions compared to uninflamed areas (Fig. 10a, b).

## Discussion

In the last decades, a considerable amount of data has been accumulated to support the hypothesis that defects in the intestinal epithelial barrier contribute to the development of IBD, even though it remains unclear which molecular events are needed to drive gut pathology. This study aimed to determine whether selective overexpression of Smad7 in epithelial cells is sufficient to induce gut damage. The motivation for this work arises from observations that Smad7 expression is upregulated in the inflamed epithelium of IBD patients, particularly at early stages in the sequence of events leading to mucosal damage [17, 37]. To address this issue, we generated mice overexpressing Smad7 selectively in the intestinal epithelium. Such animals showed segmental mucosal injury limited to the terminal ileum, likely because the method used to induce Smad7 overexpression resulted in preferential accumulation of the protein in this region [27]. Notably, the principal intestinal alterations observed in the terminal ileum of Smad7-overexpressing mice mirrored several epithelial abnormalities seen in CD, including villous blunting accompanied by varying degrees of epithelial injury, diminished mucus production, and enhanced intestinal permeability and mucosal infiltration of inflammatory cells [38, 39]. The latter was marked by a preferential accumulation of CD8^+^ T cells in the lamina propria, even though these cells were unlikely to contribute directly to epithelial injury, as their depletion did not modify the ileal abnormalities. Collectively, these observations suggest that selective overexpression of Smad7 in intestinal epithelial cells primarily promotes epithelial damage and alters barrier integrity.

To characterize the transcriptomic landscape of the ileal tissue, we next processed ileal samples from Smad7-overexpressing and control mice using the Visium platform. As expected, many genes were differentially expressed in the two groups of mice, with a marked down-regulation of genes involved in purine metabolism in Smad7TgCre^+^ mice, including *Ada*, *Slc28a2*, and *Nt5e*. Nt5e encodes for CD73, a membrane-bound glycoprotein that converts AMP to adenosine. There are several existing lines of evidence arguing for a role for CD73 in the regulation of the intestinal barrier and gut inflammation. CD73 is extensively expressed on the luminal surface of gut enterocytes [40]. Moreover, CD73-deficient mice are more susceptible to both dextran sulfate sodium (DSS)-induced colitis and 2,4,6-trinitrobenzene sulfonic acid-induced colitis than their WT counterparts [41] and are unable to resolve inflammation even after the removal of DSS [42]. Finally, an exacerbation of TNBS colitis can be induced in wild-type mice by blockade of CD73 activity with the selective inhibitor α,β-methylene ADP [42].

The factors/mechanisms regulating the cell-specific expression of CD73 are not fully understood, even though there is evidence that the CD73/*Nt5e* gene promoter contains at least one binding site for hypoxia-inducible factor-1, which mediates the hypoxia-induced CD73 expression in epithelial cells [43]. Moreover, studies in hepatic stellate cells identified two consensus SP1 motifs and one SMAD binding site, each of which was necessary for *Nt5e* gene upregulation [35]. Since SP1 and SMAD are downstream effectors of TGF-β1 signaling pathways, CD73 can likely be transcriptionally regulated by TGF-β1. This hypothesis is supported further by the demonstration that *Nt5e* RNA expression is enhanced by in vitro stimulation of mouse splenic T cells, bone marrow-derived dendritic cells, and peritoneal macrophages with TGF-β1 [44]. The data of the present work confirm and expand on these findings, as we showed that TGF-β1 positively controlled *Nt5e* gene expression in primary IECs.

Our transcriptomic data are in agreement with those published by Anzai and colleagues, showing that, in an intestinal epithelial 3D-organoid culture model of wound repair, the TGF-β1-induced migration of IECs was associated with up-regulation of a set of genes, including *Slc28a2*, a protein expressed by enterocytes and enabling purine nucleoside transmembrane transport [45].

Metabolomic profiling of intestinal epithelial cell samples obtained from the ileum of Smad7-overexpressing mice revealed lower adenosine levels compared with controls. Adenosine is well recognized for its anti-inflammatory properties, exerting direct regulatory effects on immune cell function [46]. The regulatory effects of adenosine are mediated by four G protein-coupled receptors (i.e., A1AR; A2AAR; A2BAR; A3AR), which are expressed in the gut where they deliver signals that halt inflammation following a wide range of insults [47]. In addition to its immunomodulatory role, adenosine can act directly on epithelial cells to promote barrier integrity and tissue repair. For instance, in the pulmonary epithelium, adenosine regulates mucus production and secretion; however, its regulatory functions in the intestinal epithelium remain poorly understood [48]. Here, we show that the oral administration of adenosine to Smad7-overexpressing mice enhances mucus production and attenuates the ileal mucosal damage. Although these findings do not exclude the possibility that the protective effects of adenosine are secondary to the action on additional cells other than epithelial cells, in vitro stimulation of primary IECs with adenosine resulted in a positive regulation of *Muc-2* transcripts. Taken together, these data indicate that CD73-generated adenosine signals either prevent or restrain uncontrolled intracellular pathways that would otherwise cause epithelial damage, as seen in Smad7-overexpressing mice. In this context, it is noteworthy that adenosine has a very short in vivo half-life, lasting less than 10 s, and is rapidly deaminated to inosine by adenosine deaminase [49]. In contrast, inosine exhibits a substantially longer in vivo half-life than adenosine, with an approximate duration of 15 h [50]. These observations suggest that the improvement in barrier integrity observed in Smad7 transgenic mice following oral adenosine administration is driven by short-lived local effects of adenosine on the ileal epithelium or, more plausibly, by downstream metabolites with prolonged biological activity.

A somewhat different scenario would seem to emerge from studies in SW480 cells, a colorectal cancer cell line, showing that TGF‑β1 affects the purine metabolism, through the modulation of laccase domain‑containing 1, an enzyme that converts adenosine into inosine, thus reducing adenosine levels [51]. A possibility is that such divergent results reflect the differences in the systems adopted to assess the impact of TGF‑β1 on purine metabolism (i.e., in vivo* vs *in vitro studies).

To translate our findings to humans, we assessed the expression of CD73 and Smad7 proteins in both inflamed and uninflamed ileal samples from CD patients. In the inflamed CD tissue, including the epithelial compartments, there was an up-regulation of Smad7, and this was associated with CD73 down-regulation as compared to the unaffected mucosal compartments.

This study has certain limitations. The spatial transcriptomics analysis was conducted using a single biological sample per condition, which constrains the robustness of differential expression and clustering analyses. Nevertheless, the main findings from the spatial transcriptomics analysis were independently confirmed using complementary methods with multiple biological replicates, including real-time PCR, immunofluorescence, immunohistochemistry, and metabolomic profiling. Stimulation of normal IECs with TGF-β1 activated Smad3, leading to increased *Nt5e* expression. Consistently, inhibition of Smad3 activation in Smad7 transgenic mice was associated with reduced *Nt5e* levels, suggesting that TGF-β1–mediated Smad3 signaling positively regulates *Nt5e* expression. However, it remains unclear whether Smad3 directly binds to and controls the Nt5e promoter. The major changes in the mucosa-associated microbiota of Smad7-overexpressing mice are purely descriptive and do not provide sufficient evidence to establish a mechanistic link between ileal pathology and mucosal dysbiosis. However, the finding that ileal pathology was not prevented or reversed by treatment with a broad-spectrum, bacteria-depleting antibiotic cocktail argues against a significant role for these microbial changes in the mucosal injury observed in Smad7-overexpressing mice.

Collectively, the finding that Smad7 overexpression in IECs drives gut pathology reinforces the idea that Smad7 upregulation is pathogenic in IBD. This is in line with earlier phase 1 and phase 2 clinical trials, which reported clinical and endoscopic benefits of Mongersen, an oral Smad7 inhibitor, in patients with active CD [52–56]. However, a multicenter, randomized, placebo-controlled phase 3 trial was prematurely discontinued after a futility assessment showed no efficacy of Mongersen in these patients [57]. The reasons for the discrepancies among the clinical trials remain poorly understood, although recent evidence suggests that the various batches of Mongersen used in the phase 3 study differed chemically, with some failing to downregulate Smad7 expression [58]. Therefore, new clinical studies with more potent Smad7 inhibitors are needed to further evaluate the role of Smad7 in IBD. Since Smad7 is upregulated in both epithelial and immune cells in IBD [26], selectively targeting Smad7 in IECs may be less effective than broader inhibition. Furthermore, additional research is required to determine the effects of restoring TGF-β1 signaling on intestinal fibrosis and fibrostenotic CD, given that TGF-β1 is a major driver of gut fibrogenesis [59, 60].

## Conclusion

Our data indicate that Smad7 overexpression in IECs is sufficient to alter the barrier integrity and promote ileal damage. Smad7 transgenic mice exhibit an impairment of purine metabolism, as evidenced by reduced expression of CD73, the rate-limiting enzyme in adenosine production, and diminished production of adenosine. Consistently, oral administration of adenosine enhances mucus production and largely inhibits the ileal pathology.

## Supplementary Information

Supplementary Material 1.

Supplementary Material 2.

Supplementary Material 3.

Supplementary Material 4.

Supplementary Material 5.

Supplementary Material 6.

Supplementary Material 7.

Supplementary Material 8.

## Data Availability

The data generated during this study are available from the corresponding author upon reasonable request.
