# Supplementary material for: Intestinal epithelial Smad7 drives purine metabolic dysregulation and ileal inflammation
Source: J Biomed Sci. 2026 Feb 17;33:18. doi: 10.1186/s12929-026-01224-3 (PMC12914907; doi:10.1186/s12929-026-01224-3)
Supplement: Supplementary file 1 — Supplementary Material 1. [file 12929_2026_1224_MOESM1_ESM.docx]

**SUPPLEMENTARY DATA**

**Intestinal Epithelial Smad7 Drives Purine Metabolic Dysregulation and Ileal Inflammation**

Federica Laudisi^1^, Mattia Alberto Serra^1^, Lorenzo Tomassini^1^, Massimo C. Fantini^2^, Gustavo Monasterio^3^, Ning He^3^, Eduardo Maria Sommella^4^, Angela Ortenzi^1^, Giuseppe S. Sica^5^, Francesca Zorzi^6^, Teresa Pacifico^1^, Ombretta Melaiu^7^, Cristiano De Stefanis^8^, Valentina D’Oria^8^, Carmine Stolfi^1^, Ivan Monteleone^9^, Eduardo J. Villablanca^3^, Giovanni Monteleone^1,5^.

^1^ Department of Systems Medicine, University of Rome Tor Vergata, Rome, Italy;

^2^ Department of Medical Science and Public Health, University of Cagliari, Cagliari, Italy;

^3^ Division of Immunology and Respiratory Medicine, Department of Medicine Solna (MedS), Karolinska Institute and University Hospital, SE-171 76 Stockholm, Sweden; Center for Molecular Medicine (CMM), Karolinska University Hospital, SE-171 64 Solna, Sweden;

^4^ Department of Pharmacy, University of Salerno, Fisciano, 84084, Salerno, Italy;

^5^ Department of Surgery, University of Rome "Tor Vergata", Rome, Italy.

^6^ Gastroenterology Unit, Azienda Ospedaliera Policlinico Tor Vergata, Rome, Italy;

^7^ Department of Clinical Sciences and Translational Medicine, University of Rome "Tor Vergata", Rome, Italy;

^8^ Bambino Gesù Children's Hospital, IRCCS, Rome, Italy;

^9^ Department of Biomedicine and Prevention, University of Rome Tor Vergata, Rome, Italy

**Figure legend**

**Suppl. Figure 1:** Generation and characterization of Smad7TgCre^+^ mice*.* **a.** Schematic view used to generate Smad7TgCre^+^ mice and induce Smad7 overexpression in the intestinal epithelium. **b.** Representative Western blots and densitometric analysis showing Smad7 and β-actin in intestinal epithelial cells isolated from the ileum and colon of Smad7Tg (n=3) and Smad7TgCre^+^ (n=3) mice 4 weeks after the final tamoxifen (TMX) injection. **c.** Representative immunohistochemistry images showing phosphorylated Smad3 (p-Smad3) in ileal sections taken from Smad7Tg (n=3) and Smad7TgCre^+^ (n=3) mice treated as in panel a. Scale bar: 50 µm. 5 µm (insert). **d**. Representative immunofluorescence images of TUNEL-positive cells in ileal sections taken from Smad7Tg (n=3) and Smad7TgCre^+^ (n=3) mice treated as in panel a. Scale bar: 50 µm. Data in the right graph are presented as mean ± SEM, with each point representing the number of TUNEL-positive cells per crypt, counted in four different fields per section. **e**. Representative immunohistochemistry images showing Ki67-positive cells in ileal sections from Smad7Tg (n=4) and Smad7TgCre^+^ (n=4) mice treated as indicated in panel **a**. Scale bars: 50 μm. Data in the right graph are presented as mean ± SEM, with each point representing the number of Ki67-positive cells per crypt, counted in four different fields per section.

**Suppl. Figure 2:** Representative flow-cytometry dot plots showing the percentage of CD45⁺CD8⁺ cells in splenocytes of Smad7TgCre^+^ mice following four intraperitoneal injections of a specific anti-CD8 depleting antibody (5 mg/kg), administered starting the day after the final tamoxifen (TMX) injection.

**Suppl. Figure 3:** Quality control of the spatial transcriptomics (ST) datasets. **a.** Violin plots showing the number of unique genes per spot (nFeature_RNA) and RNA copy counts (nCount_RNA) in ileal samples from Smad7Tg and Smad7TgCre^+^ mice. **b.** Schematic picture of the selection and analysis of representative areas: healthy epithelium in Smad7Tg mice (epithelium-1, -2 and -3, left panel), and healthy epithelium and damaged epithelium in Smad7TgCre^+^ mice (right panel) used to assess *Ada*, *Nt5e*, and *Slc28a2* gene expression.

**Suppl. Figure 4:** Schematic picture showing the protocol used to treat Smad7TgCre^+^ mice with adenosine via oral gavage. Mice received adenosine (10 mg/kg, n=4) or vehicle (control, n=4) every other day, starting the day after the final tamoxifen (TMX) treatment and continuing until day 28.

**Suppl. Figure 5: a.** Representative immunohistochemistry images showing lysozyme (Lyz)- positive cells in ileal sections from Smad7TgCre^+^ mice treated with adenosine (10 mg/Kg, n=5) or vehicle (n=5) via oral gavage every other day starting the day after the last treatment with tamoxifen until day 28. Scale bars: 50 μm. Data in the right graph are presented as mean ± SEM, with each point representing the number of Lyz-positive cells per crypt, counted in four different fields per section. **b.** Representative immunohistochemistry images showing leucine-rich repeat-containing G protein-coupled receptor 5 (Lgr5)-positive cells in ileal sections from Smad7TgCre^+^ mice treated as indicated in panel **a**. Scale bars: 50 μm. Data in the right graph are presented as mean ± SEM, with each point representing the number of Lgr5-positive cells per crypt, counted in four different fields per section. **c.** Representative immunohistochemistry images showing Ki67-positive cells in ileal sections from Smad7TgCre^+^ mice treated as indicated in panel **a**. Scale bars: 50 μm. Data in the right graph are presented as mean ± SEM, with each point representing the number of Ki67-positive cells per crypt, counted in four different fields per section. **Suppl. Figure 6: a.** Expression of IL-6, TNFα, IFNγ and IL-17A RNA transcripts in the ileal samples from Smad7Tg mice (n=4) and Smad7TgCre^+^ mice treated with adenosine (10 mg/Kg, n=5) or vehicle (n=4) via oral gavage every other day starting the day after the last treatment with tamoxifen until day 28. Data in the graphs are presented as mean ± SEM, with each point representing the cytokine RNA expression for a single mouse.
