# Supplementary figures and images for: Intestinal epithelial Smad7 drives purine metabolic dysregulation and ileal inflammation
Source: J Biomed Sci. 2026 Feb 17;33:18. doi: 10.1186/s12929-026-01224-3 (PMC12914907; doi:10.1186/s12929-026-01224-3)

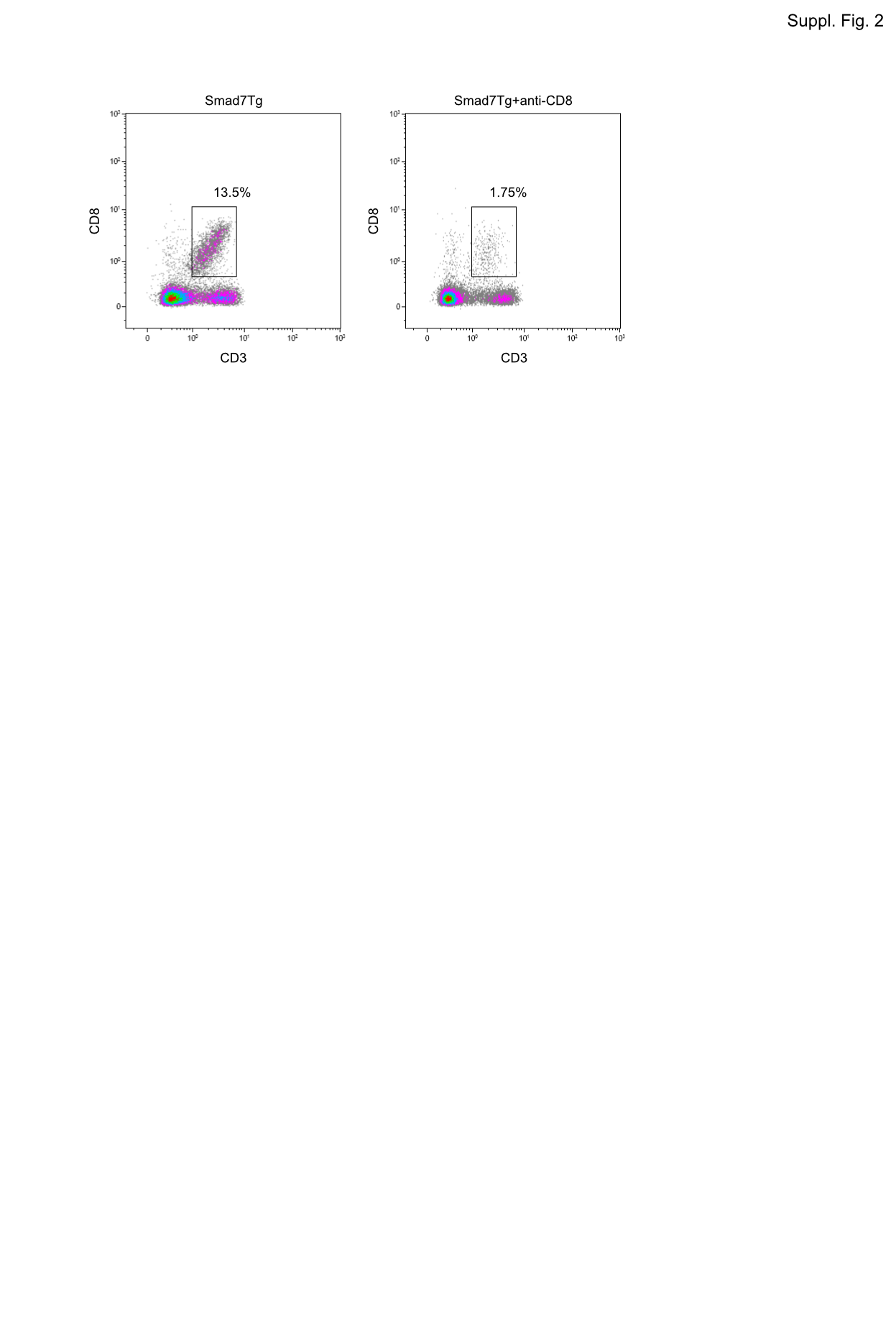

Supplement: Supplementary file 3 — Supplementary Material 3. [file 12929_2026_1224_MOESM3_ESM.png]

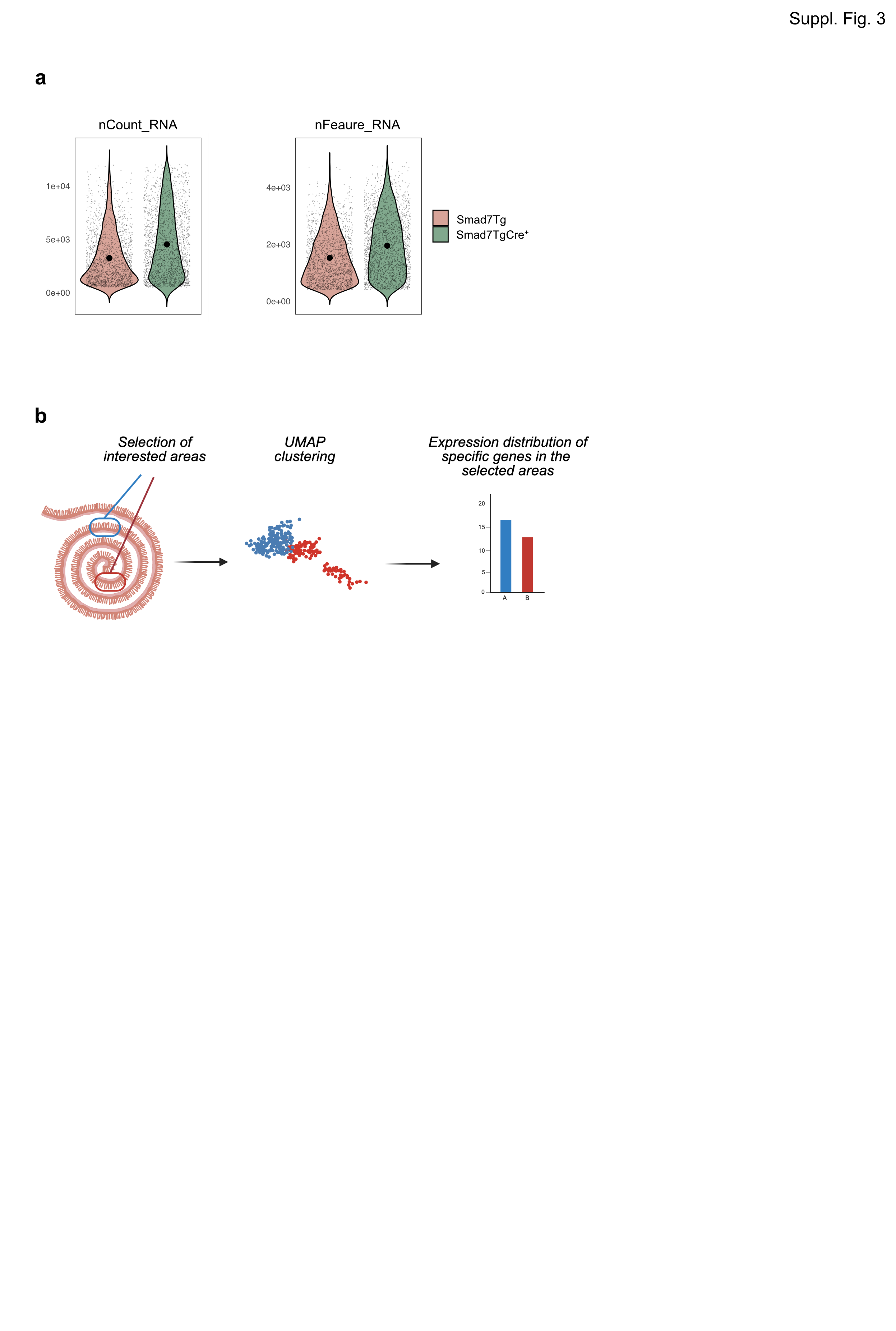

Supplement: Supplementary file 4 — Supplementary Material 4. [file 12929_2026_1224_MOESM4_ESM.png]

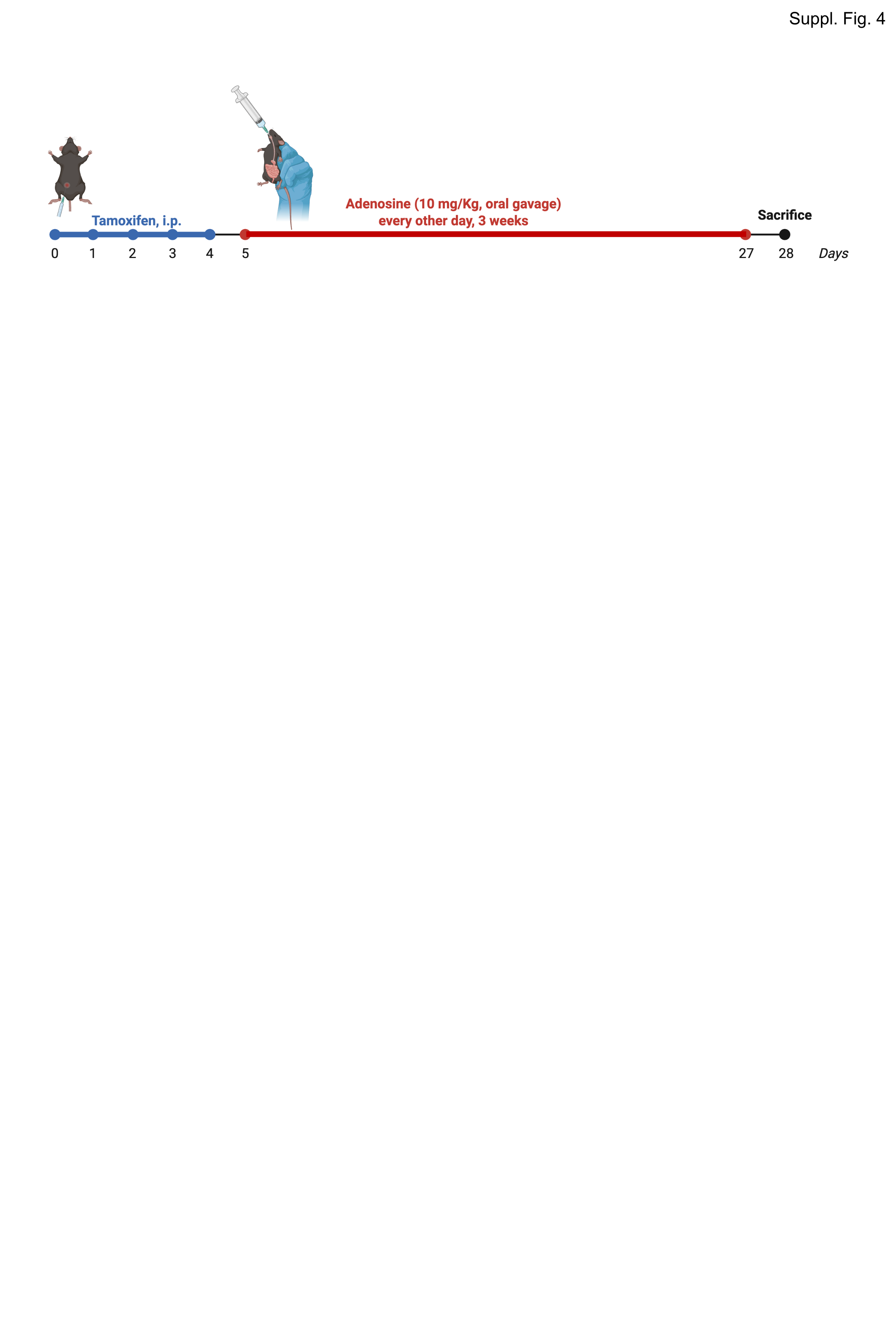

Supplement: Supplementary file 5 — Supplementary Material 5. [file 12929_2026_1224_MOESM5_ESM.png]

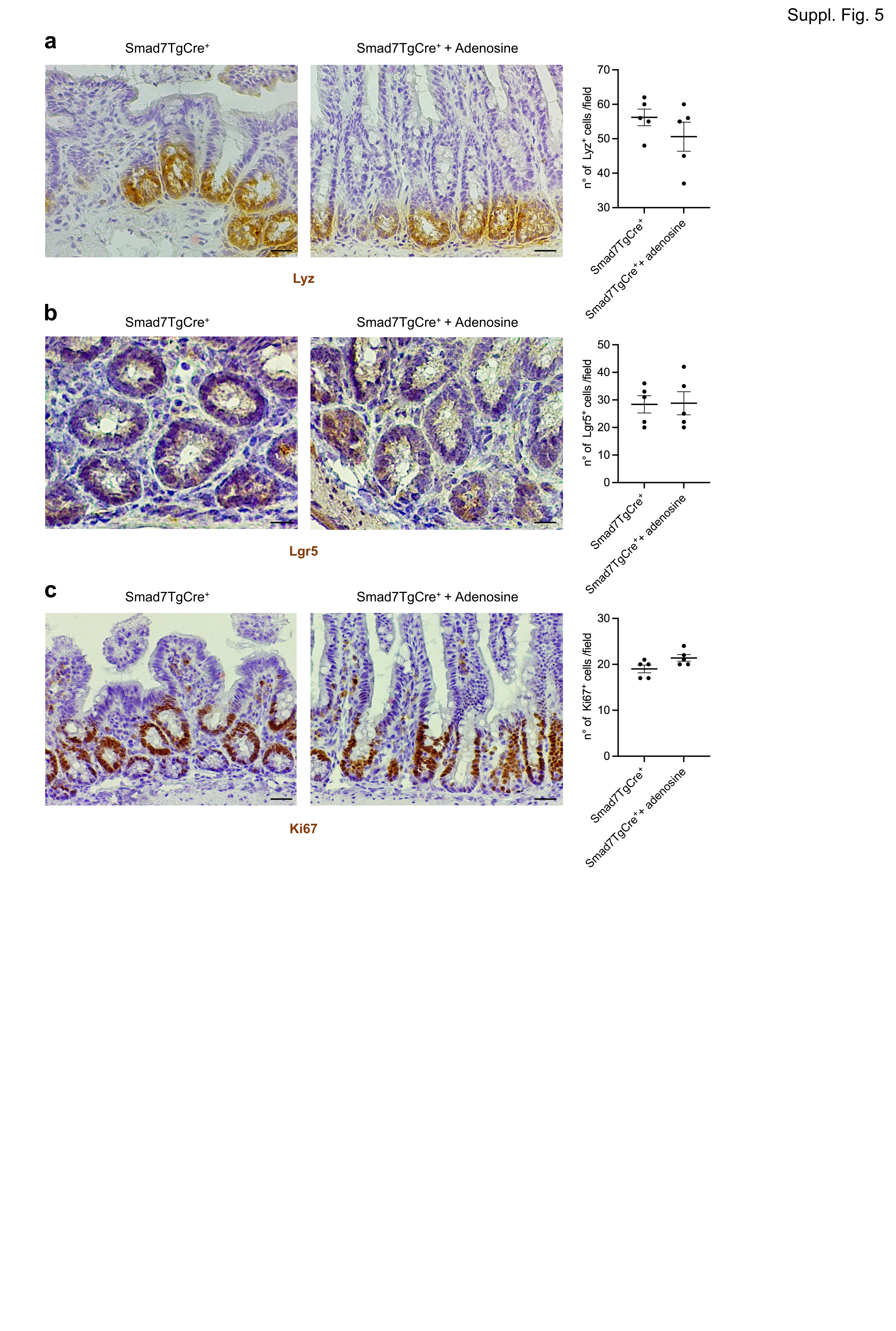

Supplement: Supplementary file 6 — Supplementary Material 6. [file 12929_2026_1224_MOESM6_ESM.png]

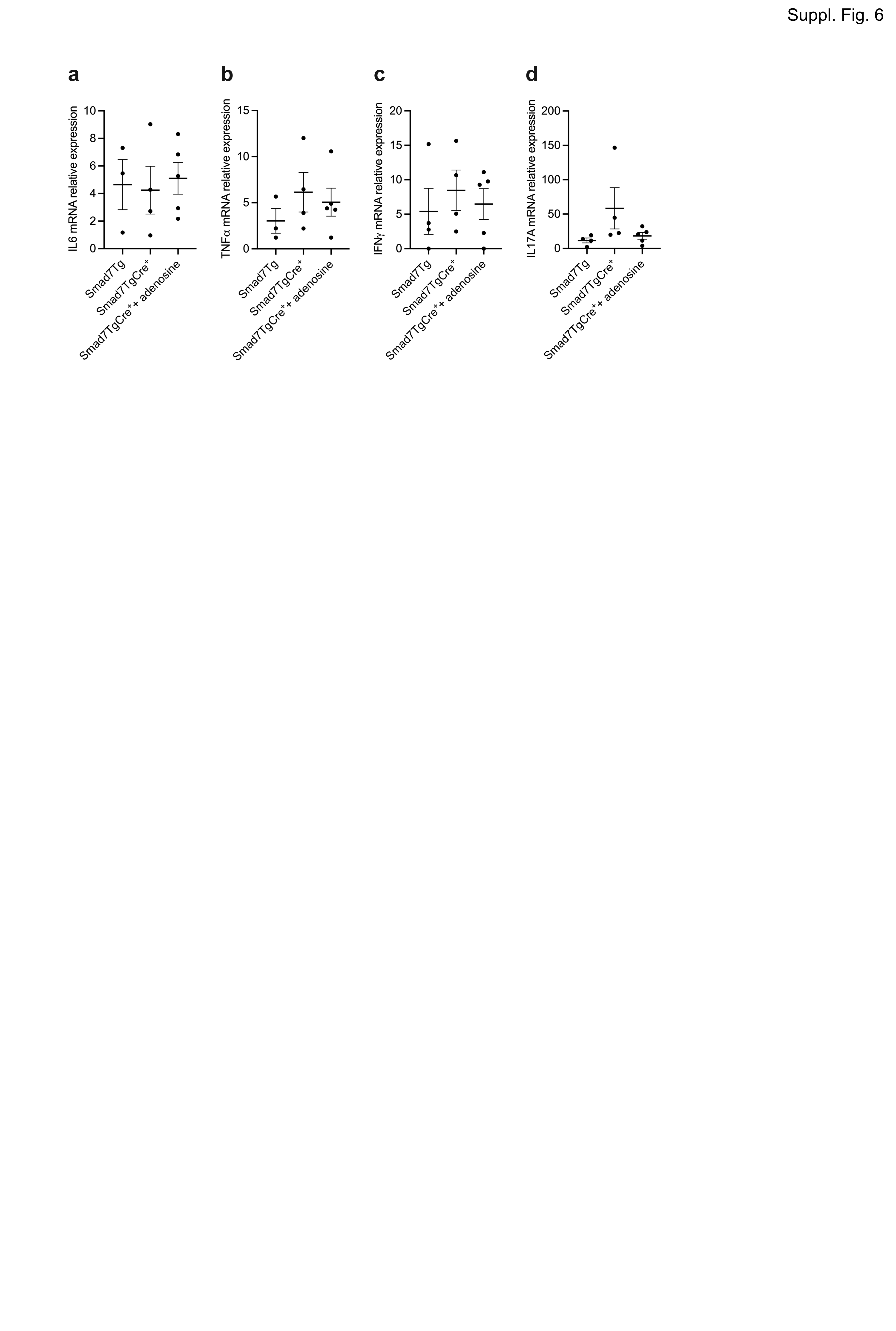

Supplement: Supplementary file 7 — Supplementary Material 7. [file 12929_2026_1224_MOESM7_ESM.png]

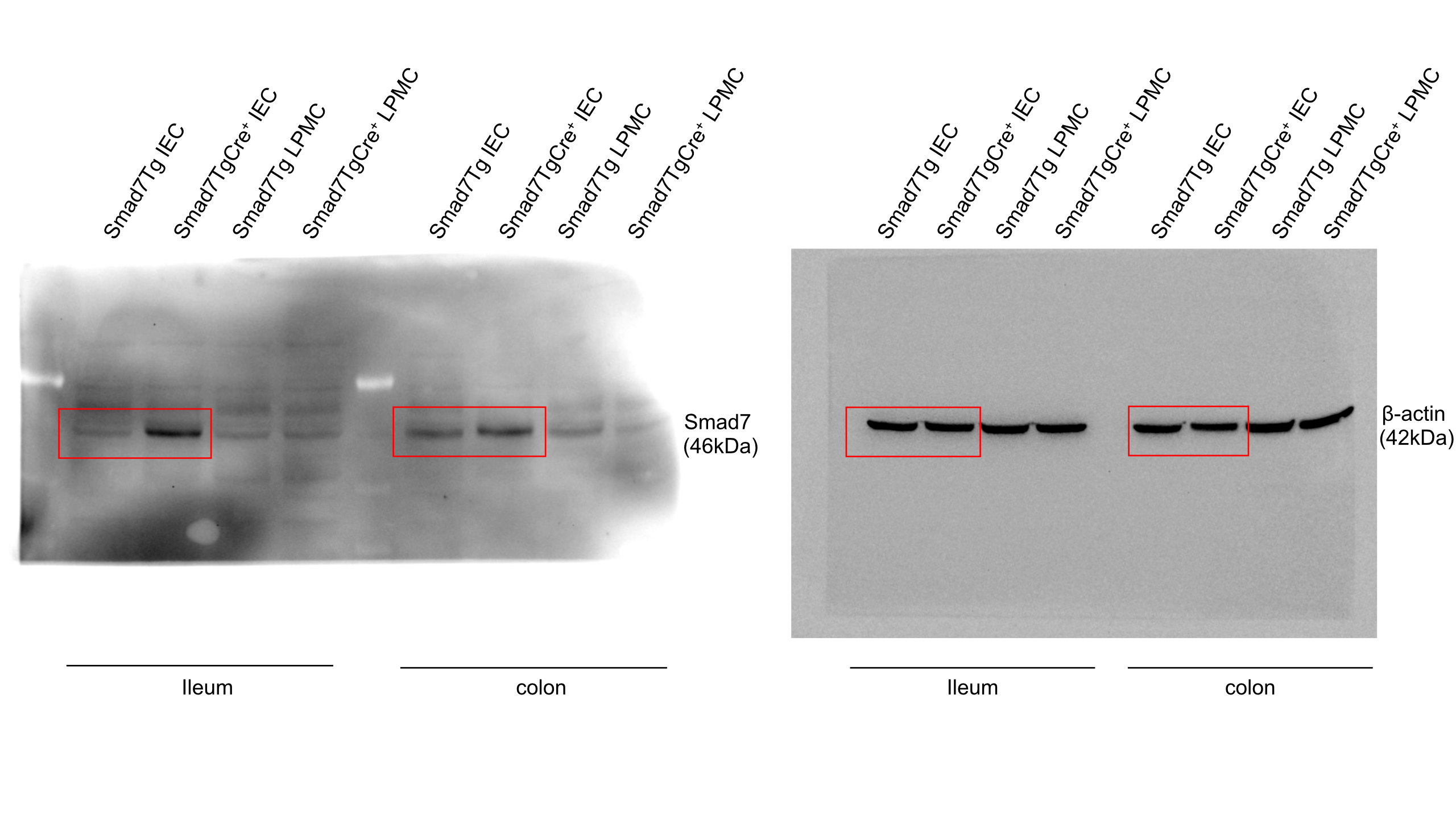

Supplement: Supplementary file 8 — Supplementary Material 8. [file 12929_2026_1224_MOESM8_ESM.png]
